# DNA Preservation in Vertebrate Samples: Insights from Forensic and Ancient Contexts

**DOI:** 10.3390/life16101601

**Published:** 2026-09-24

**Authors:** Beatrice Natale, Michal Pokorný, Daniel Vaněk

**Affiliations:** 1 Institute for Environmental Studies, Charles University, Benátská 2, 12801 Prague, Czech Republic; 2 Department of Genetics and Microbiology, Charles University, Viničná 5, 12844 Prague, Czech Republic; pokornymich@natur.cuni.cz; 3 Forensic DNA Service, Budinova 2, 18081 Prague, Czech Republic; 4 Bulovka University Hospital, Budinova 2, 18000 Prague, Czech Republic; 5 Department of Forensic Medicine, Second Faculty of Medicine, Charles University, 11000 Prague, Czech Republic

**Keywords:** DNA preservation, sample storage, forensic genetics, ancient DNA, vertebrate genetics

## Abstract

Sample preservation is a critical factor in the success of genetic analyses of biological material, particularly in ancient and forensic contexts where DNA degradation and contamination are prevalent. Analytical outcomes are influenced by multiple factors, some unavoidable, whereas others, including sample collection, handling, preservation, and downstream analyses, can be optimized. This study evaluates current DNA preservation strategies for human and non-human vertebrates, focusing on both biological samples (before DNA extraction) and extracted DNA. The goal was to identify common methodologies, limitations and challenges, and finally to propose a standardized workflow that might improve data quality and reproducibility. After evaluating a broad group of references, 202 of them were chosen as the most representative, with the idea of reporting a minimum quantity of information for each category of biological samples and preservative methods. At the beginning, a brief introduction regarding ancient DNA and forensic genetics, and an explanation about the process of DNA degradation and its related causes, explain the context. Subsequently, the main section of the review contains an analysis of the preservation methods used for each sample type, followed by extracted DNA. Some of these methods (freezing, ethanol, formalin, formaldehyde) have been traditionally used to preserve any kind of sample. Nonetheless, their limitations must be considered, especially when dealing with damaged DNA, which might require other procedures to be stored without inducing further damage. According to our evaluation, freezing and dry storage (especially at RT) remain the most reported approaches overall, even though their performance is strongly sample-type-dependent, with RT-based methods addressing freezing-related problems. This appears to apply to both biological samples and extracted DNA. Combining multiple techniques can be particularly effective, in fact the addition of liquid storage solutions is frequent. Data is insufficient for secretions, touch DNA, and swabs, even though dry storage appears quite common; however, among them, urine and saliva are comparatively better characterized. Considerations regarding the phase prior to storage (sampling, handling and transportation) and post-storage (downstream applications) are also discussed, since they will eventually affect the choice of the storage solution. It was not possible to define a particular difference among storage methods for human samples and other vertebrate animals, apart from the fact that human studies are much more present in the literature.

## 1. Introduction

Researchers continue to optimize DNA workflows, storage techniques, and conditions to ensure high-quality DNA. One of the main challenges in genetic analysis is DNA degradation. DNA degradation is a fundamental process that occurs in both living and dead organisms [1]. The main implications of this process concern PCR failure, inhibition, and miscoding lesions [2]. In fact, DNA damage is generally considered a major obstacle in genetics, especially when dealing with challenging samples that, if left unprotected for a long time, have been subjected to compromising physical and chemical conditions. This is especially true for forensic and ancient samples. Genetic analyses of forensic and ancient DNA pose significant challenges due to the presence of low-quality, highly fragmented, and chemically damaged samples. These characteristics complicate analyses or, in some cases, hamper them.

Mitochondrial DNA (mtDNA) and nuclear DNA (nDNA) are equally employed in several applications. The main difference lies in the advantage of mtDNA in being present in higher copy numbers in the cells, simplifying the extraction from highly degraded samples and the amplification processes [3,4]. Moreover, nDNA is more susceptible to rapid degradation [5], making its use in degraded samples particularly challenging, with the risk of producing less reliable DNA profiles compared to mtDNA [1]. On the other hand, relying on nDNA can be crucial if mtDNA fails; when dealing with animal samples, mtDNA is usually employed in species identification applications but, since it is inherited only from the maternal lineage, it does not always lead to an accurate species-level identification.

Post-mortem DNA degradation is inevitable due to the chemical instability of DNA itself [6], and it continues despite the preservation methods employed, which can only slow it down. Nevertheless, in certain conditions, nucleic acids can persist [7]. Time plays an important role in nucleic acid degradation, as degradation rates increase over time, and depend on the environmental conditions to which samples are exposed [8]. However, time alone is insufficient to drive DNA degradation, since preservative conditions exert a stronger influence [9], as evidenced by the long-term preservation of archeological remains. Environmental factors can independently induce DNA damage, but because they typically occur simultaneously at forensic and archeological retrieval sites, their effects are mutually amplified. Additionally, each factor’s contribution depends on exposure conditions and the sample matrix. When conducting ancient and forensic analyses, these factors should all be considered [10]. Although degradation is inevitable, favorable environmental conditions can allow DNA to persist even in very ancient specimens. Moreover, careful selection of an appropriate preservative method, based on sample type, degradation rate, and downstream analysis, can significantly improve DNA recovery and data quality.

DNA damage also results from improper sample handling and storage, and from late protection against environmental factors [11]. Expected DNA damage includes fragmentation, base modifications, contamination that leads to DNA mixtures, and, in the worst case, total degradation [11,12]. DNA degradation is also tissue-dependent, reflecting differences in body anatomy and tissue morphology [11], an aspect that will be explored in depth.

In this review, we aim to highlight methodologies used to preserve biological samples of human and non-human vertebrates and extracted DNA in ancient and forensic genetic analyses. The aim is to find common methodologies, identify existing gaps, and propose a workflow for selecting the most suitable storage strategies, based on sample type, origin and desired downstream application. Because the references are broad, it was not feasible to assess the efficacy of preservation according to a single criterion. Thus, the evaluation of good storage conditions is made in accordance with a variety of aspects of DNA analysis, mainly including PCR success and STR completeness, but also considering the DNA degradation level and, to a limited extent, DNA recovery. More specifically, high DNA recovery is not a direct measurement of successful storage, since it does not necessarily lead to successful amplification and sequencing. At the same time, higher yield after a specific storage method is still a metric in support of the suitability of a certain method, and it is a widespread criterion in the literature references regarding storage conditions. Nonetheless, the analytical endpoint of the references considered is specified to highlight whether, for example, high DNA recovery effectively leads to successful downstream analysis. The main points that we want to investigate are: I. Preservative methods for biological evidence before DNA extraction. II. Preservative methods for extracted DNA. III. Differences between forensic and ancient samples. IV. Differences between humans and other vertebrates.

### 1.1. Ancient DNA

Ancient DNA (aDNA) studies are highly important in the fields of archeology and archeozoology [8]. Zooarcheology is the analysis of animal remains from the past. Most aDNA applications in animals concern domesticated animals because this knowledge is applied in agronomy [13]. However, some of these studies also address wild species, which is highly valuable because reconstructing the demographic history of past wild populations helps to understand the present genetic diversity and contributes to conservation efforts [14]. Zooarcheological studies are also important for understanding the relationship between ancient animals and humans, helping to elucidate hunting, scavenging, domestication, and husbandry practices [13]. Early aDNA studies focused on phylogeny and phylogeographic patterns [15] applied to extinct and extant species. This was due to the technological obstacles posed by non-high-throughput technologies [15]. Only limited amounts of DNA were analyzable, and usually of mitochondrial origin, thereby limiting the obtainable information about evolution [14]. Moreover, the first types of samples used in aDNA studies were bones and teeth, given their high prevalence and reliability.

A major breakthrough in ancient genetics was the application of Next Generation Sequencing (NGS) in the first decade of the 21st century [16], which enabled the efficient recovery of highly fragmented aDNA molecules, without requiring long intact DNA templates for PCR. The first applications of NGS to aDNA studies focused on extinct species, beginning with Neanderthal fossils and mammoth bones [13].

Ancient DNA is already degraded [13], making ancient genetic studies challenging. DNA typically degrades into short fragments after hundreds or thousands of years because cellular repair processes are active only in live cells [6]. The main processes responsible for aDNA damage include DNA fragment length, polymerase stalling, and the appearance of miscoding lesions [17]. In particular, the most abundant modification is the deamination of cytosine to uracil, which causes C→T (and G→A) substitutions and overall artifactual GC-AT mutations [15]. This event is related to time and on-site temperature [13,18]. Nonetheless, a major challenge in aDNA analysis is contamination. Ancient DNA is often contaminated with mixtures of endogenous ancient DNA, environmental/microbial DNA and exogenous modern human DNA. Each of them requires a different approach for authentication strategies. The distinction of aDNA sequences from modern human contamination relies on several aspects, including differences in the DNA sequence, deviations from expected ploidy, and typical DNA degradation patterns such as short fragment length and terminal cytosine deamination [19]. Depending on the study design and the available data, a range of different approaches can be incorporated, including the assessment of negative controls, molecular duplication and library complexity, mitochondrial or nuclear contamination estimates, sex-chromosome-based methods, mapping behavior evaluation and other quality controls. In addition, the proportion of endogenous DNA recovered from a sample is an important parameter in aDNA studies; it is used in the authentication process to separate endogenous molecules from contaminants. However, endogenous content usually constitutes less than 1% of the sequenced reads, increasing costs and efforts necessary for shotgun sequencing [20].

### 1.2. Forensic Genetics

DNA analysis in forensic investigations has become a fundamental practice. In forensics, the time of sample collection is very important, since it has been demonstrated that DNA degradation increases with the length of the post-mortem period [21], and the same holds for the presence of microbes that deteriorate the body [7].

The primary aim is to analyze short tandem repeats (STRs) along the nuclear genome [22]. Over time, animal DNA has also been used as evidence in several applications, such as species identification, individual identification, parentage testing, and food fraud [23]. In crimes, animals can be victims, witnesses, or perpetrators, just like humans [23]. Wildlife forensics is a distinct sector, a growing scientific field situated between wildlife conservation and applied forensic science [24]. The techniques used in animal forensic genetics have been developed from human forensic genetics, which relies on robust databases and well-established procedures. An interesting application of animal forensic samples is the assessment of the geographic origin of confiscated animal parts, such as elephant tusks, described by Ogden et al. [24] as the ‘reproductive population of origin’. The problem with animal databases is that they are less detailed and require improvement, particularly given the wide variety of species [25]. Contamination is a risk not only in ancient genetics but also in forensic science, especially for low-template DNA samples, because exogenous human DNA can compromise analytical results [26]. Retesting is particularly important in forensic science [27]; therefore, long-term storage of samples is essential and maybe be required by legislation, retention policies or post-conviction review requirements, depending on the jurisdiction.

Beyond serving as an obstacle, DNA degradation studies help forensic investigators to obtain additional information, including estimation of the post-mortem interval, time since deposition, forensic archeology, environmental forensics, comparative genomics, forensic anthropology, and mixture interpretation [1]. All of this is possible thanks to fundamental characteristics of DNA, such as its stability [1].

## 2. Mechanism of DNA Degradation

Understanding the mechanisms of DNA degradation is fundamental to all genetic applications [2]. Biological material continuously encounters biotic and abiotic factors that accelerate and intensify its degradation. In living organisms, continuous cellular processes such as apoptosis and autophagy contribute to the regulated degradation of DNA [1]. In contrast, after cell death, DNA rapidly degrades because cellular mechanisms that normally repair DNA damage are inactivated [28]. DNA degradation is a cascade mechanism that can arise from exogenous or endogenous sources and can involve enzymatic activity.

Enzymatic degradation, primarily led by nucleases, is among the first factors to start post-mortem DNA fragmentation [2]. In particular, nucleases act differently on DNA: endonucleases cleave phosphodiester bonds and consequently form smaller fragments, while exonucleases trim terminal nucleotides [1]. Non-enzymatic degradation can occur alongside enzymatic degradation or shortly after. It is slower but persistent, and it is caused by various factors, including oxidative reactions, chemicals, the environment, and physical forces [29].

The following section focuses on environmental factors, which are particularly relevant in forensic and ancient contexts, where samples are exposed to outdoor conditions for long periods of time.

### 2.1. Environmental Factors of DNA Degradation

The external environment heavily influences the constant chemical modifications of DNA, which occur even under optimal conditions [1].

#### 2.1.1. Temperature

The main effect of elevated temperature on DNA degradation is an increase in chemical reaction rates, including hydrolysis and oxidative damage mediated by reactive oxygen species (ROS).

As a general rule, biomolecular preservation of biological samples is better in cold environments than in hot ones [30] because low temperatures reduce enzymatic/microbial activity and slow chemical reaction rates. This explains how ancient DNA can be preserved in permafrost for thousands of years. In contrast, warm climates are associated with rapid increases in DNA damage, which accumulates over time [31].

Moreover, high temperatures, together with time, modulate cytosine deamination according to a regular thermal-age model [18]. However, DNA degradation in cold environments can also occur, likely due to other simultaneous factors that cause the damage. High DNA degradation in winter may be caused by moisture, as suggested by Piggott and Taylor [32] for fecal material. Opinions on the effect of freezing on DNA integrity vary widely; what matters is that the low temperature used to preserve DNA is constant; otherwise, DNA structural disruption may occur. As early as 1996, Höss et al. [33] reported that a 20 °C reduction in temperature leads to a 10–25-fold-lower rate of nucleotide base degradation.

Nonetheless, DNA preservation depends not only on the nominal storage temperature but also on temperature stability and the frequency of freeze–thaw cycles.

#### 2.1.2. Microorganisms

Microbial degradation can occur either because conditions support microbial growth or because of poor sample storage. Bacterial presence varies by body part and tends to shift from aerobic to anaerobic species in later stages [34]. Bacteria degrade organic compounds to obtain energy through fermentation or respiration.

#### 2.1.3. Humidity

Increased humidity can initiate and accelerate DNA degradation via hydrolysis. Hydrolysis separates the sugar component from the base due to the break in the N-glycosidic bond [35]. Although hydrolytic reactions can occur in both hydrated and dry states, the absence of water significantly reduces the rate of DNA hydrolysis compared with hydrated conditions.

Water generally supports microbial growth, and hydrolytic and oxidative DNA damage. Rainfall and moisture significantly contribute to DNA degradation in samples collected in outdoor environments [32,36]. However, DNA damage also depends on the amount of contamination in the water and the length of the DNA fragments, with longer fragments being more susceptible to cleavage in wet environments [37].

#### 2.1.4. Radiation

UV radiation is divided into three types: UV-A (315–400 nm), UV-B (280–315 nm), and UV-C (<280 nm) [9]. Although UV-C radiation is the most energetic and highly genotoxic, stratospheric ozone largely absorbs solar UV-C before it reaches the Earth’s surface. Therefore, human exposure at ground level is predominantly to UV-A and UV-B radiation, which represent the main sources of solar UV-induced DNA damage [7,38]. UV radiation produces pyrimidine dimers (CPDs), responsible for causing the main cytotoxic lesions, as well as 6-4 photoproducts (6-4PPs), which induce prominent perturbations in the DNA structure. However, it is not completely understood if the influence of 6-4PPs is detrimental to DNA. UV radiation also damages DNA by producing free radicals, such as ROS, which generate oxidized nitrogen bases and other oxidative damage [38]. Thus, UV exposure on DNA can progressively result in initial pyrimidine dimer formation and oxidative base damage, ultimately reducing DNA integrity and amplifiability. As a result, ss and ds breaks appear, especially in dehydrated samples [10].

Other forms of radiation may similarly hamper the integrity of DNA. For example, X-rays also adversely affect DNA amplification, possibly constituting an issue for the ancient samples that are regularly treated with radiation before collection. However, in a recent article on bone samples, the authors could not find a damaging association between radiations and DNA amplification, also suggesting that when precautions are taken, the risk is manageable [39]. The same principle applies to UV irradiation, which is commonly used for sterilization and can induce DNA damage, making it suitable for simulating UV exposure in samples.

#### 2.1.5. pH

pH variations influence DNA in different ways, like denaturing the double helix and altering base-pair interactions [40]. In particular, acid and alkaline environments speed up DNA degradation, while neutral and near-neutral (low-alkaline) environments contribute to preserve DNA [2,41,42]. pH also influences microbial growth [1].

#### 2.1.6. Oxygen

Reactive oxygen species (ROS) generated in the presence of oxygen can oxidize DNA bases and induce single- and double-strand lesions [1]. Oxygen availability can also influence aerobic microbial activity [1,41].

#### 2.1.7. Soil

Soil constituents, such as nutrients, organic matter, water, and air, can damage DNA [2]. Among microorganisms regularly found in soil, more than 70% contain nucleases capable of destroying DNA molecules [11].

## 3. Review Methodology

This work is intended to be a narrative review. Considering the broad scope of the topic, the search was limited to a selected number of representative references.

A literature search was performed between October 2025 and June 2026. The platforms used for the search were Google Scholar, ScienceDirect, and PubMed. No restriction was applied to the publication date of the retrieved articles, as this would have excluded several older references that we considered highly relevant for this topic. No language or date restriction was applied during the search. Several old studies were considered essential for the scope of the review, particularly for the mechanisms underlying DNA degradation or early findings in storage methods. The first and second authors selected the articles independently. The main keywords used for the search were “forensic AND DNA AND preservation”, “ancient AND DNA AND preservation”. To narrow the search to only humans or animals, the following keywords were also used: “animal AND DNA AND preservation” and “human AND DNA AND preservation”. References were selected based on their relevance to the topic discussed, including DNA degradation mechanisms, forensic genetics, and ancient DNA analysis, empirical data, and methodological comparisons. Studies lacking such content, or unrelated to DNA preservation, were excluded.

After a first round of searching, the findings were compared to remove duplicates, and discrepancies in selection were resolved by discussion until consensus was reached. Additional publications were selected from the references of the articles already selected or intentionally searched in the databases to ensure enough representative examples for each section. In this context, “representative” references are defined as those selected, among a set of comparable studies addressing the same sample type or preservation method, to ensure that the 202 references collectively cover the broadest possible range of sample types, taxonomic groups, and preservation strategies discussed in the manuscript. In case of sample types for which information was missing, for example hair, a first search was conducted combining the sample-specific keyword with “forensic” and/or “ancient”, for example, “animal AND hair AND preservation AND forensic/ancient”. If the search did not give results, it was broadened by removing the forensic/ancient qualifier, for example, “animal AND hair AND preservation”.

When the necessary information was not available from peer-reviewed articles, other sources such as technical reports and communications were included if consistent with the topic and the findings reported in the rest of the selected literature.

## 4. Preservation of Biological Evidence Before DNA Extraction

Adequate sample preservation is crucial for maintaining high quality and preventing nucleic acid degradation [12,43,44,45,46,47]. General recommendations include eliminating light and contaminants and reducing exposure to temperature, water, and oxygen [48].

Generally, hard materials contain little DNA that is difficult to extract, even though it is more stable and less fragmented. Conversely, soft tissue contains more DNA, which is easier to recover but more prone to decomposition and fragmentation.

Different specimens require different preservation techniques, since the stability of nucleic acids is also influenced by the tissue type [21,49] and taxonomic group. Traditionally, birds’ and mammals’ skins and bones have been preserved dried, while reptiles and amphibians have been preserved in ethanol or formalin [48].

Thus, freezing, cryopreserving, ethanol, formalin, and formaldehyde have been among the most used methods for sample preservation. Freezing may not perform as well as other preservatives [50], but may preserve DNA sufficiently to produce complete allelic profiles and avoid inducing relevant levels of degradation within 35 months [51]. The problem with freezing, especially for forensic and ancient samples, is that it can be problematic in the field and during transport, and it requires large storage space.

Ethanol and formalin (in particular, 10% buffered-neutral formalin (BNF)) are commonly used in museum archives to preserve samples such as organs and tissues, and their use in forensics is also common. Absolute ethanol and water-free acetone have also been used to preserve forensic tissues, yielding high-molecular-weight DNA with yields comparable to those obtained under freezing conditions [21], as well as 70% ethanol [52]. However, even when DNA extraction yields an acceptable amount, amplification and sequencing may still fail, which is the problem usually encountered in the end. The 70% ethanol concentration is widely used; it has traditionally been used for tissue storage, and is effective for preserving, but it is not necessarily good for DNA. Effectiveness depends greatly on the tissue and its water content, but ethanol generally affects DNA integrity, especially after extensive preservation. Preservation in formalin can impair PCR performance, even though this issue can be overcome by using buffered formalin. Formaldehyde can markedly increase DNA degradation even after brief exposure [53]. However, the use of both ethanol and formalin is connected to health risks. Because of its carcinogenicity, formalin can be replaced with a GAF (glyoxal-acid-free) solution, which ensures high yield and high-quality DNA and could, in the future, replace formalin for long-term tissue fixation in the forensic field [54].

This section describes the methodologies used to preserve specimens in the laboratory, as appropriate conditions are essential for extended storage. Collection/recovery studies are discussed separately when operationally relevant; their outcomes are not treated as evidence of preservation efficacy unless storage suitability is directly evaluated. Nonetheless, because studies in this field differ in matrix, storage duration, temperature and primary endpoint (e.g., DNA yield, degradation indices, STR success, or sequencing metrics), comparative statements throughout this section are qualified by the specific conditions under which they were obtained, rather than presented as generalizable rankings.

### 4.1. Soft Tissues

- Humans

The preservation of tissues for disaster victim identification (DVI) can be quite challenging because of the remote areas where these events might occur and the fact that, due to the absence of electrical power and the difficulty in employing portable refrigerators, reagents used at room temperature (RT) are usually the only possible solution. Michaud and Foran [50], in an effort to identify a suitable storage method for human tissues from DVI, used pig tissues stored with several preservatives, and found that a DMSO solution performed best among the methods tested, based on the amplification success of the largest amplicon (642 bp) over 6 months, even for the most degraded samples [50]. For short-term storage, isopropanol and ethanol also provided effective preservation, whereas desiccation was the least effective [50]. DMSO has been repeatedly recognized as an effective preservative for long-term storage, with the particular advantage that it works well at high temperatures. This is particularly important for DVI in tropical areas, a condition that Allen-Hall and McNevin [55] sought to replicate by storing human-donated tissues at 35 °C with eight different reagents for short-term storage (28 days) in both solid and liquid form. Preservation in NaCl, DMSO, EtOH-EDTA, DNA Genotek Tissue Stabilizing Kit (DNA Genotek, Ottawa, Ontario, Canada), and DNAgard^®^ successfully produced complete genotype profiles for solid preservation, whereas only DNAgard^®^, DMSO, and DNA Genotek produced full profiles of the liquid preservative. However, TENT yielded the highest overall DNA quantity from the liquid preservative, but this was accompanied by most partial STR profiles [55]. In another example, DMSO preserved muscle tissue for 4 weeks at both RT and 37 °C, whereas NaCl and 70% ethanol successfully preserved DNA after 2 weeks at 37 °C and up to 4 weeks at RT (15–20 °C) [56]. Other studies investigating DVI in tropical climates have shown that modified TENT buffer delivered variable performance depending on the study and endpoint considered. It yielded a higher DNA quantity and more complete STR profiles than FTA cards in one study on decomposing tissues [57]. However, modified TENT produced mostly partial profiles despite high DNA yield in another study on fresh tissue in tropical conditions [55], and similarly Sorensen et al. [58] retrieved high yield from decomposing tissues over 3 months at 35 °C, but with the majority of resulting STR profiles (58%) only partial. Moreover, Worrapitirungsi et al. [59] found modified TENT more suitable for fresh tissues and the short term, assessing the low potential of preservation with it only after 2 months. Instead, DNA/RNA Shield™ (Zymo Research, Irvine, CA, USA), a nucleic acid stabilization solution that works at RT, was recognized as an effective preservative for long-term storage and was better suited to degraded samples, based on degradation index values that remained stable over time in both fresh and decomposed tissues, unlike modified TENT [59]. Alternatives for long-term storage include the Oragene^TM^ DNA self-collection kit (DNA Genotek, Ottawa, ON, Canada) and Lysis, Storage, and Transportation (LST) buffer, which were tested for the preservation of muscle tissue for a period of 12 months at RT (24.2 °C) [60]. The Oragene^TM^ kit outperformed the LST buffer in DNA yield, but DNA quality was not significantly different between them, resulting in STR profiles in both cases. The optimal yield from the Oragene^TM^ kit was achieved with 500 mg of muscle, whereas the LST buffer is more effective for preserving low amounts of tissue (<100 mg). DNA degradation began after 6 months in both cases [60]. Another example of tissue storage in DVI comes from Birne et al. [61], who preserved 44 tendons in NaCl for up to 12 months, with an average DNA recovery of nearly 100%, producing complete STR profiles in 34 samples. Untreated tendons, on the other hand, had an average DNA recovery of 3% and, unlike NaCl-preserved tendons, showed macroscopic signs of decomposition after 6 months [61]. Caputo et al. [62] also used NaCl for DVI, demonstrating that soft tissues stored at RT (temperature not specified) in a dry salt environment for over 1 year showed no signs of damage.

The use of salt as a tissue preservative is an ancient technique that has been employed in ancient Egypt and the Chinchorro culture of South America for artificial mummification [63]. Shved et al. [63] replicated the desiccation step of mummification on a lower limb of an adult female by storing it in natron salt and monitoring temperature, relative humidity, and pH. Skin and muscle tissue samples were collected over several time periods, totaling 322 days. DNA fragmentation and DNA lesions were observed, with an earlier decline in muscle than in skin (11 days post-mortem versus 38 days post-mortem). However, the overall results showed that even at the end of the experiment, there was no allelic dropout of STR markers up to 350 bp, and that, in general, the increase in degradation registered on the last day was not significant. Therefore, salt desiccation can be considered an effective method for long-term preservation of human tissue [63]. Holmes et al. [57] made a different attempt, preserving muscle tissue at RT (temperature not specified) for 6 months with FTA^®^ cards, typically used for blood and liquid samples, compared with TENT. FTA^®^ card preservation showed poor downstream performance, with 53% of samples being PCR-inhibited and 31% showing no detectable alleles [57]. PCR inhibition may reflect the presence of inhibitors in the extracted samples, instead of being necessarily related to degradation. In general, FTA^®^ cards can preserve DNA from post-mortem (PM) tissue samples, but the outcome depends on tissue conditions, such as the level of decomposition [64]. Notably, morphological preservation of soft tissue does not guarantee DNA survival: brains preserved for ~80 years via saponification in a Spanish Civil War mass grave yielded no recoverable DNA despite intact gross and histological structure, indicating that the conditions favoring tissue preservation are not necessarily favorable to DNA integrity [65].

- Other animals

The DESS principle of preservation is based on the deactivation of metalloproteins such as DNases [47]. Seutin et al. [43] found that DESS-preserved avian tissue samples, stored at RT for up to 24 weeks, yielded a DNA quantity and quality similar to samples cryopreserved at −70 °C and −20 °C over the same period, whereas 70% ethanol showed very low yield and significant degradation after 6–11 weeks [43]. Oosting et al. [47] evaluated the effect of DESS and >99.5% ethanol on the initial stages of DNA degradation in fish (*Chrysophrys auratus*), confirming higher DNA preservation with DESS (moderate degradation rate after 3 months) compared to ethanol (high degradation rate after 24 h). Combining DESS with refrigeration at 5 °C yielded the best result observed across the whole experiment, with over 50% high-molecular-weight DNA retained after 3 months [47].

According to Kilpatrick [44], the DNA could be extracted from mammalian tissue even 2 years after extraction at RT. DMSO buffer can also be employed; it demonstrated the capacity to preserve pig tissues, as stated earlier [50].

The employment of Longmire’s Lysis Buffer ensured a high integrity level of DNA stored for three to four weeks at 60 °C, even though there were no reproducible amplification results [66]. Consequently, it is not feasible to assess its effectiveness on downstream applications. Camacho-Sanchez et al. [67] demonstrated that even a home-made nucleic acid preservation (NAP) buffer could successfully preserve DNA on post-mortem rat tissues. The NAP buffer preserved DNA better than 95% ethanol and cryopreservation for a period of 7 weeks. This is attributed by the authors to the presence of EDTA in the NAP buffer, which, as confirmed by other studies [44], increases the preservation rate when combined with ethanol or used in other solutions like DMSO or Longmire buffer. However, DNA degradation was detected just after 6 h post-mortem, but large DNA molecules persisted at high concentrations even in non-preserved muscle samples left at RT (temperature not specified) for up to 1 week [67]. Solid vertebrate tissues can also be stored with NaCl [47]. DNA degradation was also investigated in mammalian cells, and showed a reduction with the usage of trehalose [68]. The effectiveness is increased when trehalose is coupled with freeze-drying [68].

#### Blood

- Humans

Blood is probably the most frequently analyzed biological sample; therefore, its changes during storage at different temperatures and reagents have been intensively studied. Storing blood in a freezer can be an effective way to preserve it, but the temperature should be constant.

To assess DNA degradation, DNA blood samples were stored for 83 days at 37 °C, RT (21 °C), and 4 °C, and analyzed every 3 days [69]. No additional preservatives were added; only EDTA was used as an anticoagulant. Interestingly samples stored at 37 °C showed higher DNA recovery. However, this result was not correlated with a correspondent lower degradation index (DI). In fact, despite evident degradation patterns in STR analysis, the study did not show a direct correlation between storage techniques and DNA degradation, or success in downstream applications. Regarding the yield, the authors hypothesized that higher temperatures dry the samples, thereby limiting the negative effects of humidity, and that inhibitory substances decompose faster at higher temperatures [69].

Outdoor crime scenes may require the collection of blood mixed with soil, which may have different storage requirements than pure blood. For that reason, a blood–soil mixture (1 g of soil, 1 mL of blood, 4 mL DNase/RNase-free water) was stored in three different conditions (RT (25 °C), 4 °C, and −20 °C) for different periods of time (2, 4, 6, 10, and 12 weeks) [70]. The most effective method was −20 °C, with a registered degradation index below 1 after 12 weeks. RT storage was also a favorable option, because, at week 4, the degradation index was less than 1 and continued to rise through week 12 when it was moderately degraded (ranging from 1 to 10). At 4 °C the index rose immediately after 2 weeks, and at week 8, no DNA was detected [70]. Regarding chemical preservation, 4-month-old blood samples were stored at RT (temperature not specified) with FDL (Forensic DNA Laboratory) buffer which yielded ~2.6 µg, and with sodium citrate, which yielded ~2 µg [71]. The extraction method influenced the outcome in this case, as did the sample type, since FDL buffer testing on saliva yielded a much lower yield than another chemical [71].

Menstrual blood is important evidence in forensic investigations, as it helps obtain STR profiles for individual identification. Menstrual blood was collected from 30 healthy females every 2 h for 48 h and kept at 25 °C. In total, 24 complete STR loci (23 autosomal loci and the amelogenin) were successfully typed. Even though a diminished quality of the profiles was observed over time, DNA could still be recovered and typed at 48 h sampling [72].

When storing blood samples, it is necessary to consider the hemoglobin inhibitory function, which may interfere with subsequent PCR reactions. Poor blood preservation may lead to autooxidation of Hb and cause DNA damage. For example, wet samples stored in plastic containers yielded the worst serological and spectroscopic outcomes for Kaur et al. [73]. Dry blood samples are well preserved and more suitable for producing high-quality spectroscopy of Hb and DNA [73].

In the forensic context, FTA^®^ cards are routinely used for blood preservation and have been proven to be effective, compatible with useful field techniques such as direct PCR [74], and especially convenient in the long term [75]. FTA^®^ cards are composed of a cellulose membrane treated with a specific patented chemical formulation. It is imperative to be cautious about the quantities of blood put on FTA cards because overloading can introduce excessive amounts of hemoglobin, a known PCR inhibitor, consequently reducing the card preservation effect. When this occurs, FTA^®^ cards may necessitate additional purification steps to ensure successful amplification, for example, using a cost-effective purification buffer (10 mM Tris-HCl [pH 8.0], 10 mM EDTA, 50 mM NaCl, 1% SDS) [76], or reducing PCR input to a capacity that is permitted by FTA classic cards. However, these procedures are downstream applications and not actually improvements for preservation.

Bloodstains are another interesting source of DNA. Hara et al. [77] stored bloodstains on gauze for 20 years at different temperatures (RT, 4 °C, −20 °C, −80 °C), blood samples in −20 °C and −80 °C for the same period, and fresh blood. Integrity was assessed by the ratio of long and short DNA fragments (129:41 bp and 305:41 bp) [69]. The optimal integrity level required for long-term storage was achieved with fresh blood and storage at −20 °C and below. DNA from bloodstains did not prove to be effective for long-term storage over 20 years; thus, the authors suggest considering it only for preliminary testing [77]. However, there is proof of successful preservation of bloodstain DNA at RT for short periods of time ranging from a few weeks [78] to 1.5 years [79], and also for up to one year at high relative humidity (RH) (93%) at RT or 35 °C, though only for one month under more extreme temperatures (55–65 °C) [80]. This data is also consistent with the observation that DNA in dried bloodstains can survive for months or years at ambient temperature [81]. A particular but feasible condition of bloodstain analysis is when they are deposited on concrete. Under this condition, amplification yields were lower because concrete contains many ions that act as PCR inhibitors. Thanks to the trapping function of FTA^®^ elution cards, they can be washed with ddH2O before use for analysis to remove concrete debris. On two types of concrete (pavement, bricks), 0.5, 1, and 2 μL of blood was applied, allowed to dry for 24 h, and sampled onto FTA^®^ elution cards placed under a 0.8 kg weight [82]. STR amplification success on FTA^®^ cards (~92%) was considerably higher compared to swabbing (~17%), and samples from concrete bricks showed a decreasing tendency according to the initial blood sample amount [82].

- Other animals

Vertebrate blood samples have usually been preserved using buffer solutions such as EDTA, SDS, and DMSO [48]. Although there are no specific examples in the literature about FTA cards used to preserve aDNA or forensic DNA from animal blood, successful examples from another context exist and they should be mentioned to underline the feasibility.

FTA^®^ cards are widely used in conservation genetics, especially for ease of use in the field, ease of transport, and high-quality DNA. Smith and Burgoyne [30] demonstrated that PCR amplification can be performed on highly degraded DNA from blood stored on FTA^®^ cards by omitting the deproteinization wash and therefore increasing the number of cycles. Among species, the usage of FTA^®^ papers for avian blood has to be well thought out because bird blood contains more DNA; therefore, dilution before use is recommended [30]. In fact, filter papers were not recommended for avian blood by Di Lecce et al. [83], whose survey of research experiences instead indicated better perceived outcomes with lysis buffer, ethanol, freezing, or RNA*later*^®^. RNA*later*^®^ is a stabilizing solution that suppresses RNAse inactivity and can be used at RT. This product can also be used to preserve DNA, even though interference with DNA extraction has been demonstrated on tissues [50].

Limitations connected to downstream analysis were also investigated in mammals. DNA extracted from FTA cards was found to be adequate for traditional genetic applications (e.g., microsatellites) but not for genomic applications requiring high-quality, high-quantity DNA [84]. Even so, and regardless of the species, FTA cards are particularly convenient for the field collection of blood [85], as well as GuHCl, especially for large quantities of blood from large animals.

### 4.2. Hard Tissues

Hard tissues, like bones, nails, and teeth, can better resist the effects of environmental factors [86]. Those can be distinguished as mineralized and keratinized tissues.

#### 4.2.1. Mineralized Tissues

##### Bones

- Humans

By comparing bones stored at −20 °C, RT, dehydration/freeze-drying, lysing buffer, or 96% ethanol without the addition of additional additives, it was found that for long-term preservation (1 year), it is better to freeze the bones, but for shorter-term storage (6 weeks), it is preferable to place samples in ethanol [87], based on DNA concentration (full STR profiles were obtained regardless of the preservation method). However, storing old bones under freezing conditions for longer (10 years) could lead to a 5-fold decrease in bone DNA yield [88].

Preservation in a stabilizing medium is a possible alternative to freezing. Results from the preservation of human bones in NaCl suggest that under non-ideal conditions, it is possible to store bones in salt at RT (20 °C) for 30 days without affecting DNA quality [89]. In support of this, Afrifah et al. [90] reported a real forensic case in which a full STR profile was obtained from a femur recovered from a naturally salty burial environment, demonstrating the feasibility of DNA recovery under such conditions, although this only constitutes an observational case rather than a controlled experiment. Another important factor is the form in which the bones are stored, and powdering is usually effective. Bones preserved in powder form contained a significantly higher DNA yield (0.062 ng/mg) than a whole piece (0.03 ng/mg) after 10 years of preservation at −20 °C [91]. Powder also provided good results when stored at RT (23–28 °C) for up to 3 years, producing 17 solid STR profiles out of 21 [92].

Another influencing factor is the correlation between bone parts and the amount of DNA. Analysis of bones of different ages (1 to more than 200 years) showed that the highest DNA yield was obtained from the outer parts, while inner or middle parts yielded less [93]. On the contrary, DNA from the middle parts of the bone has the highest quality and more fragment length specificity than others [93]. Also, the amount of DNA in human bones differs according to the body site (e.g., arm), and even within the same body site (e.g., humerus, radius, ulna). This was confirmed by analyzing the quantity of DNA from three buried individuals for 4 years. Starting from approximately 0.2 g of bone powder per sample, the average highest DNA concentration was obtained from the foot (with some exceptions due to higher recovery from bones of the lower torso), while the lowest was from the arm. This also correlates with STR recovery (94.15% for foot versus 73.10% for arms) [94]. However, the results of this study might be biased by the sampling method, which consisted of drilling to obtain powder, which might have damaged the DNA through factors such as increased heat. Different results were recorded for the three individuals, but the variations in burial depths unevenly influenced the decomposition processes [94]. Interestingly, DNA analysis from the inner ear portion of the petrous bone through NGS has been suggested as a successful technique for burnt human bone remains, yielding 14.6–66.9% endogenous DNA even in degraded forensic cases [95].

In ancient analysis, the petrous bone, the hardest and most dense mammal bone, is recognized as a valuable source of DNA [96,97,98]. Compared to teeth, it showed better performance in terms of DNA content, although this result is biased by a specific group of teeth samples that were not well preserved [99]. Moreover, Hansen et al. [99] correctly noted that this result does not indicate an absolute preference for petrous bone over teeth, though both sample types are well-suited for aDNA analysis. Heat is known to be one of the damaging factors in DNA degradation, but there is proof that DNA can be extracted and amplified in some cases; however, the precise temperature that leads to the survival of nucleic acids is still unknown, since different experiments detected different thresholds in bones, like 800 °C [100] or 250 °C [101].

- Other animals

Ancient animal bones are a precious source of information for evolution studies. They are also a valuable source of DNA, since they have a higher amplification rate than hide [3]. An important challenge with this type of sample retrieved in archeological sites is the cooking process the bones underwent in the past, which causes DNA damage and might be evidenced from external signs of discoloration, friability, and morphological changes [102,103,104]. Ottoni et al. [104] noted that DNA preservation is highly variable, but it generally depends more on the organic (collagen-associated) fraction of the bone, rather than on the degree of mineral alteration. However, the cooking process is not always detrimental, as Ottoni et al. [104] were able to extract and amplify DNA from thermally damaged bovine bones, suggesting that heating does not necessarily alter mtDNA recovery and that thermally damaged bones should not be automatically excluded from the analysis. Analyzing fish bones could be particularly advantageous for the retrieval of aDNA, according to the hypothesis that the lack of bone remodeling in archeological fish bones could contribute to endogenous DNA preservation [105].

As with humans, the choice and availability of different types of bones have a great influence on the outcome of genetic analysis. Skull samples are the best source of good-quality DNA yield in mammals, even for low copy numbers [3].

Moreover, major factors influencing the recovery of good levels of endogenous DNA from bones include retrieval sites and pre-extraction protocols. Some authors suggest balancing the need for pre-extraction treatments (such as digestion or washing) of ancient animal bones [105], since they may increase the amount of DNA but reduce sequencing library success. Others, like Pruvost et al. [45], completely avoided those treatments on fresh unwashed bone samples and found that, compared to washed and long-stored (old) samples retrieved from the same site, fresh unwashed samples yielded substantially more amplifiable DNA. Amplification succeeded in 5 of 8 fresh bones versus 0 of 11 old bones; across a broader dataset spanning multiple sites, fresh bones contained on average six times more DNA than old, treated ones [45]. Regarding forensic context, bones may also be retrieved from water, introducing additional factors that may influence DNA preservation. Thornton et al. [106] tested DNA recovery from waterlogged porcine ribs and scapulae and found that, although the overall DNA quantity did not differ significantly between bone types or locations, DNA quality (according to the degradation index) declined faster in scapulae than in ribs, and faster in the river than in the lake. Despite this decline, sufficient DNA was recovered to generate an STR profile after more than one year of submersion (up to 4000 accumulated degree days).

In a proper laboratory, the literature reports examples of ancient bone samples immediately frozen at −20 °C after collection [45], or sun-dried and stored in cardboard boxes [45], or stored at −80 °C [106].

##### Teeth and Tusks

- Humans

DNA preservation in human teeth depends on intrinsic characteristics, such as the type of tooth and its composition, age of the individual, and presence of pathologies. Nonetheless, teeth have been proven to be particularly resilient, possibly even more than bone tissue, due to the presence of protective structures like mineralized enamel, cementum and alveolar bone [107].

Sampling from the cementum layer is known to optimize the DNA yield [108,109]. Higgins et al. [110] observed nDNA yields 1–2 orders of magnitude higher in cementum compared to dentine, while mtDNA was mostly observed in root dentine. After 1, 2, 4, 8, and 16 months of teeth being buried, more than 50% of full STR profiles were observed in every period only in the cementum; coronal dentine had less than 10% full STR after one month, and root dentine had less than 10% after two months [110]. Another factor influencing DNA yield in teeth is the difference between adult/non-adult, permanent/deciduous, and fully/not fully developed. Statistically, a higher yield is found in adult teeth (1.3 versus 0.18 ng DNA/g tooth), permanent teeth (0.89 vs. 0.06 ng DNA/g tooth), and fully developed teeth (1.14 vs. 0.34 ng DNA/g tooth) [111]. Moreover, some substances found on teeth might exhibit stabilizing or disruptive properties. Pokšāne et al. [112] investigated the role of copper patina in DNA stabilization of human bones and teeth from five different cemeteries. The endogenous aDNA proportion (a shotgun-sequencing-based authentication metric, not a genotyping success rate) was found to be lower for patinated teeth (0.009–0.045%) than for the unpatinated (1.182–2.210%), while for the bone, no difference was noticed (0.275% and 0.208–0.231%). This shows that copper patina does not increase DNA stability but can degrade it. In contrast, apatite stabilizes DNA molecules. DNA adsorption on apatite substrate helps maintain its integrity, simulating processes observed in hard tissues (bones, teeth).

Adsorption is reduced at low temperatures and promoted at RT, while the addition of phosphate ions promotes DNA release [113]. Dental calculus, formed by mineralized dental plaque [114], is a particularly valuable source of aDNA for its higher DNA yield and resistance against microbes and environmental damage compared to dentin [115]. However, most of the DNA recovered is of microbial origin [115]; therefore, the use of this matrix is still unclear [107].

Regarding storage techniques, Rubio et al. [116] stored various types of teeth (canines, molars, premolars) without preservatives at RT (temperature not specified) for 0 to 10 years. A significant difference in DNA yield was observed only between fresh samples and older ones (more than 2 years), but no difference was observed between older samples (2–10 years); therefore, it was suggested that the degradation process occurred mainly during the first 2 years [116]. DNA was quantified in 87.5% of the samples, except for two teeth stored for 5 and 10 years [116]. Terada et al. [117] showed that the use of a commercial DNA stabilizer (Allprotect™) significantly increased the DNA quantity in intact tooth samples stored at RT for up to 180 days (*p* = 0.008), but provided no benefit for refrigerated or frozen samples, or for dental pulp under either temperature condition. Given the high price of the reagent, the authors suggest its use is justified when RT storage over an extended period is unavoidable [117].

- Other animals

The literature on the preservation of animal teeth and tusks is scarce, but some examples and general recommendations exist.

Rahmat et al. [118] compared freeze-drying of molar and premolar pig teeth against normal air drying. Average DNA yield from freeze-drying was approximately two times higher than from air-dried teeth (6.42 ± 2.64 versus 3.06 ± 1.51 ng/µL), but the difference between drying methods was also dependent on the type of teeth (premolar/molar) and the type of tested DNA (nDNA/mtDNA) [118]. In the case of burning, DNA concentration in animal hard tissues (both bones and teeth) decreases with increasing exposure time [119]. Tusks should be preserved under stable conditions of RT and relative humidity in the range of 45–55% [120]. Stressful environmental conditions can, in fact, cause cracking in tusks [121].

#### 4.2.2. Keratinized Structures

Interest in the analysis of keratinized structures is growing rapidly. Thanks to the outer layers of keratin or collagen, these types of samples are more amenable to preservation and less susceptible to contamination [122], even under harsh conditions.

##### Hair

Hair is generally not considered a good source of DNA. However, it can represent a valuable source of DNA in both ancient and forensic genetics [123]. A higher yield is usually obtainable when the hair follicle is preserved [124]. In case of shed hair, nDNA is particularly difficult to recover, but endogenous mtDNA has a good capacity for survival even in particularly degraded samples [123,124,125].

- Humans

The most common storage technique for hair is to store it dry at RT, preferably with a desiccant in tubes or paper envelopes [48] although the efficacy of this approach has not been systematically quantified. In contrast, when hair is exposed to water rather than stored dry, as may occur when hair evidence is recovered from an aquatic environment, DNA degradation can occur rapidly. To investigate DNA degradation in hair, hair with roots was removed from volunteers and colored with DAPI for nuclei visualization, then washed with PBS and stored in spring water for 2 h to 240 h [126]. The amount of DNA was significantly lower after 72 h, and almost complete degradation occurred at 240 h. However, this trend was not consistent, as after 168 h, the decrease in reference hair roots was minor, and STR profiles were better than those after 72 h. The authors suggested that it might be due to stochastic effects arising from the usage of a small number of hairs. Moreover, some roots were not totally degraded after 240 h, and it probably depends on the hair stage [126]. The average completeness of STR profiles was 100%, 17.5%, 58%, and 7.7% at specific sampling times (2, 72, 168, and 240 h) [126].

There are inconsistencies about the influence of washing hair, since this practice is sometimes discouraged [127] or encouraged [128]. In general, hair is particularly persistent as a sample, even when the bodies are in an advanced state of decomposition [129]. In fact, this type of sample can also be useful for recovering other types of traces, such as blood and semen, in which DNA could be detected after 3 months of exposure to outdoor conditions [129].

- Other animals

Animal hair is usually more commonly employed for forensic DNA than for ancient DNA. This is because hair is very scarce among historical specimens, and DNA is usually fragmented in hair shafts [130]. However, one advantage of using hair is that it is easier to clean and decontaminate. The concentration of DNA extracted from 150-year-old horse hairs [130] was negatively affected by temperature and humidity, as well as by the cleaning step, even though purity was not damaged in any way. Amplification was successful, mostly for samples with a concentration of 100 ng/µL; therefore, the authors suggested optimizing the amount of sample material [130]. Also, Amory et al. [128] were successfully able to obtain complete STR profiles from hair plucked directly from Siberian mummies. An important aspect of hair is that its suitability for molecular analysis depends more on the depositional environment than on the time since the hair was deposited, as stated by Tridico et al. [131] and previously affirmed by Bengtsson et al. [132]. Silica desiccation resulted in amplification rates broadly comparable to −20 °C freezing over a 1-year storage period, although outcomes varied according to amplicon length and target type [133].

##### Nails, Claws, Hooves, Scales, Feathers

- Humans

Nails are particularly resistant to post-mortem degradation and environmental influence [134], making them a reliable sample for complex genetic analysis. While nails can be transported refrigerated (4–8 °C) [134], RT (temperature not specified) storage is effective enough to produce successful STR profiles even after a decade [135]. Piccinini et al. [135] achieved parenting testing with matching STR profiles from nails in a case where the corresponding bone sample from the same body failed to yield any result despite the use of multiple extraction protocols. The authors hypothesized that the improved DNA preservation in nails might be related to the burial position of the hands, which limited their contact with the rest of the putrefied tissues.

- Other animals

The part of the ancient feather that is most commonly sampled is the calamus, located at the base [122]. However, Rawlence et al. [136] showed that DNA can also be recovered from distal feather components, including the rachis and barbs. Feathers are important evidence in wildlife crime casework. Interesting recommendations on their preservation come from several authors in the conservation biology context. First, the use of high-quality feathers collected as soon as possible following the molting process is fundamental to generate high-quality DNA [137]. Feathers are stored properly when kept in a cold, dry, dark environment. This is because direct sunlight, humidity, and high temperature can compromise the efficiency of DNA analysis [46]. Vili et al. [46] recommend sampling the feathers from the superior umbilicus of the shed feathers. Attention should be given to the size of the fragments analyzed by PCR, as smaller and medium-sized fragments exhibit a higher rate of PCR success and are particularly suitable when there is a high level of DNA degradation [46,138].

The literature on the preservation of other types of keratinized structures is scarce, but in general, they are not usually well preserved in the archeological context [122], even though they are frequent in wildlife forensics and conservation. An example comes from successful microsatellite amplification from fish scales preserved in absolute ethanol (at 4 °C or −80 °C) or in 4% formaldehyde (temperature not specified), as well as from air-dried scales stored at RT [139]. However, DNA yield decreased progressively across these conditions, from ~35–41 ng/mg for fresh/ethanol-preserved scales to ~19 ng/mg for air-dried scales and to ~9.6 ng/mg for formaldehyde-preserved scales [139]. Pangolin scales are typically stored in plastic bags at RT during transportation for international illegal trade, and this storage approach might also be used in the laboratory. It has been demonstrated that DNA can still be detected in traces from the plastic bag itself containing the scales [140].

### 4.3. Secretions

#### 4.3.1. Feces and Urine

- Humans

After preserving six volunteer urine samples (three males and three females), 2 mm FTA^®^ card punches were selected by Srisiri et al. [141] as the most suitable size for storage. With an ideal minimum volume of 3 mL of urine for males and 2 mL for females, the protocol enables the production of enough DNA for 15-loci STR profiling [141]. Complete STR profiles were obtained only after an initial centrifugation of the samples at 4000× *g* 30 min, since higher speed might have damaged cell membranes [141]. This protocol was later improved by reducing the minimum urine volume to 1 mL [142]. RT is not suitable for urine storage without any protectant, but +4 °C and −20 °C are effective storage temperatures, since their STR typing results are similar [143]. The possibility of preserving DNA urine at +4 °C was already demonstrated in 1993 [144] for long-term storage, but with attention to removing contaminants (e.g., bacteria) before storage, since they can inhibit PCR. Cold temperatures can be used with some effective reagents, as demonstrated by Zhang et al. [145], who stored urine samples in EDTA at −80 °C. They determined that the most effective concentration for the Chinese population is 40 mM EDTA, based on the mean STR loci detection rate between males and females [145].

The recovery of exfoliated cells from fresh human stool has been reported to be on the order of millions of cells per gram [146], providing enough DNA for future analysis unless the sample is too diluted. Coprolites are not widely used in routine archeological studies [147], in part because they are difficult to identify. However, when this occurs, coprolites do not need to be treated with hydrochloric acid or hydrofluoric acid prior to analysis, unless they were recovered from sites with a high content of silicon oxides or calcium carbonate. It is recommended to make a longitudinal cut, retain one half for future analyses, and rehydrate the other half in a 0.5% aqueous sodium phosphate solution [148].

- Other animals

In the context of ancient samples, dung is usually neglected because it is more difficult to recognize, and if recognized, it is also challenging to identify the species [149]. Even though the samples are not forensic or ancient, a useful example of animal fecal preservation comes from Murphy et al. [150], who found 90% ethanol to be an effective preservative for brown bear (*Ursus arctos*) dung specimens, among other methods such as DETs buffer, silica-drying, and oven-drying. On the other hand, dung samples from Ursids were also tested by Wasser et al. [151], who instead identified silica preservation as more efficient than freezing, freeze-drying, ethanol and other buffers, according to DNA amplification outcomes. According to Murphy et al. [150], the discrepancy between the studies might be caused by the fact that freeze-drying from Wasser et al. [151] was performed before extraction, removing moisture, and the desiccation instrument used by the two studies were different.

#### 4.3.2. Saliva

- Humans

The quantity and quality of DNA extracted from saliva depend on the component under analysis. In the forensic science and biomedical fields, DNA can be extracted from whole saliva collected with a swab, or from the supernatant or the pellet obtained after centrifugation.

The Oragene^TM^ self-collection kit was specifically developed for the storage of saliva. It successfully enables DNA extraction and amplification even after several months [152] or years [153] at RT. Self-made FDL buffer is a valid alternative for preserving saliva samples for years at RT. It enabled visualization of the DNA band on agarose gel electrophoresis even after 8 years [154]. Garvin et al. [155] used drying collection tubes to collect and preserve saliva, and evaluate DNA recovery. In particular, active drying tubes (forensiX; production discontinued) in nylon or cotton were compared to passive drying swabs (Sarsted, Nümbrecht, DEU) for a total period of 14 days. The forensiX nylon swabs were completely dried after 4 h, while the cotton ones were 90% dried after 6 h. Sarstedt tubes took 29 h to completely dry. Saliva DNA yield from forensiX tubes (95%) outperformed Sarstedt (12%), and the same happened in terms of DNA degradation rate, and microbial growth. These results support the idea that drying speed is crucial to maintain recoverable DNA, and passive drying systems are not suitable to satisfy this condition [155]. However, the authors state that a test for STR profile was not conducted, given the high quantity of nDNA already obtained according to DNA recovery.

In forensic contexts, saliva samples are also regularly stored on FTA cards. An example comes from human saliva stored on FTA cards for up to 11 years, with DNA concentrations ranging from 0.1 to 2771 ng/µL [156]. However, although enough DNA was retrieved for downstream analysis, the level of DNA degradation affected the STR profiles’ quality, especially for a degradation index (DI) above 2.12, which required adjustment of the PCR input.

- Other animals

One approach to using animal saliva in the forensic context is to analyze saliva at kill sites to investigate predator–prey interactions. It has been traditionally applied to livestock and wild ungulate predation, a context with a high quantity of samples present. Peelle et al. [157] used this technique to identify the predators of a small prey species, the snow-shoe hare (*Lepus americanus*), by analyzing DNA left on carcass remains and radiocollars at kill sites. Predator saliva was collected with foam swabs for buccal collection and stored in a lysis buffer solution, allowing RT storage. DNA from any species present was successfully amplified from all kill sites, but predator DNA was identified at 58.5% of sites (31/53), with success rates not differing significantly between sites with minimal remains and those with more remains [157]. Interestingly, the choice of lysis buffer comes primarily from logistical reasons, since the resulting amplification rates were comparable to those obtained with silica-based drying preservation. A delay of a few days in saliva sampling on the carcass does not necessarily affect the outcome of the analysis [158], but, since the outcome depends on several factors, it is better to proceed with the sampling as soon as possible. Salivary DNA deposited on carcasses is subjected to a period of degradation that occurs in a non-defined time frame dependent on the specific environmental conditions. Some studies record signs of diminished genotyping success in the first 12–24 h [159] or in the following 24–48 h [160]. Additional studies support the successful recovery of predator DNA saliva on carcasses, but without assessing DNA degradation time [161,162].

### 4.4. Swabs

- Humans

The success of genetic analysis from swabs depends on several factors, including tissue type. Bathrick and Davoren [163] used an accelerated aging model at 37 °C and 50 °C for up to 548 days to assess long-term storage of human blood, saliva, semen, and vaginal samples. Only the vaginal cell samples showed a severe decrease in DNA yield during storage, while slight degradation was observed in blood and saliva samples; saliva was the least stable and produced moderate-quality STR profiles.

The choice of moisturizing agent also matters, and it can be, once again, tissue-specific. Lacerenza et al. [164] compared several preservation methods to collect whole blood, luminol-treated blood, saliva, semen, and mock skin stains. Water yielded significantly more DNA than ethanol in whole blood and semen, whereas ethanol and RNA*later*^®^ yielded significantly more DNA than water in skin samples. However, these differences in yield were reflected in STR profiling success only for skin, where ethanol and RNA*later*^®^ produced significantly more complete profiles than water (>98% vs. 71%); for all other tissue types, full STR profiles were obtained regardless of the moistening agent used, despite the differences observed in DNA yield. The authors [164] recommend active drying of swabs at crime scenes, as freezing can improve analysis results but is not always feasible.

- Other animals

Findings from Martin et al. [165] on buccal swabs from anurans in different case studies suggest that freezing yields the highest DNA recovery among the tested techniques. However, to overcome freezing-related problems, silica-drying is also a good alternative and is preferable to ethanol, which, on the other hand, may be better than a non-alcoholic buffer [165]. In general, drying is particularly common and advantageous compared to freezing or the use of liquids when dealing with remote field swabs [158], but the outcome of DNA preservation is also species-dependent [166].

### 4.5. Touch DNA

The literature regarding storage methods for touch DNA is scarce; therefore, a comprehensive evaluation of storage conditions comparable to those for other DNA sources is not currently possible to the best of our knowledge. Nevertheless, collection methods are relevant, because materials such as swabs and FTA cards can serve as both collection and storage substrates prior to DNA extraction.

- Humans

Stabilizing touch DNA might be challenging due to possible, but not necessarily, low template levels. Using a lysis buffer as a stabilizing agent for touch DNA can be more efficacious than distilled water. Based on DNA profile quality, the use of lysis buffer led to greater DNA recovery and stability compared to distilled water; samples stored in ddH2O showed significant degradation after 24 h across all tested temperatures, whereas those in lysis buffer showed markedly less degradation, remaining largely intact up to 50 °C [167]. A separate methodological consideration concerns the collection step itself. Here, the FTA^®^ paper-scraping method is a promising approach for collecting touch DNA primarily by improving DNA recovery. In particular, a statistically significant difference was detected in DNA recovery between double swabbing and FTA^®^ paper scraping, with higher yield obtained by FTA paper, and allowing half of the sample to have enough yield (1 ng) for STR analysis [168]. However, among samples with sufficient DNA, double swabbing showed a nominally higher mean percentage of STR allele recovery (99%) compared to FTA paper (91%), though this difference was not statistically significant [168].

- Other animals

An interesting application of touch DNA analysis is its application in animal forensics for the recovery of human DNA. This has been explored in several cases, through the combination of cell staining and direct PCR [169], with DNA swab analysis and subsequent STR profiling [170].

## 5. Preservation of Extracted DNA

In forensic and ancient genetics, it is more common to store whole samples in various preservation agents [52,58,156]. Preserving extracted DNA is problematic due to the adhesion of DNA molecules to polypropylene or polyethylene tubes, which reduces DNA yield [171]. However, preserving extracted DNA is also relevant. Two main reasons are: (i) Ancient and forensic samples are usually in small amounts and considering the nature of destructive DNA extraction that is usually performed, especially on some types of samples, the DNA sample is the last source of information, so it must last. (ii) It can be a convenient strategy to store mostly extracted DNA and minimize or completely avoid sample storage, especially in freezers [48]. This is important because DNA samples are much more stable than tissues from which DNA is extracted [48].

### 5.1. Physical Preservation

- Freezing and Cryopreservation

Freezing has been considered the method of choice for a considerable amount of time because enzymatic and chemical activities decline at low temperatures. However, in some circumstances, several factors may lead to the selection of an alternative method, such as spatial constraints, the absence of electrical power, and transportation complexity [84].

Regardless of the extraction and preservation method, DNA extracts are highly sensitive to freeze/thaw cycles. The higher the number of freeze/thaw cycles, the more DNA is degraded, with greater susceptibility for large-sized DNA [172]. Smaller molecules around 30–40 kb in size are particularly resistant [172]. Interestingly, the extraction method appeared to have little effect on freeze/thaw-induced DNA degradation, whereas higher DNA concentrations exerted a protective effect against it [172].

For low- and ultra-low-temperature freezing, −20 °C and −80 °C are the most used. Lower temperatures (cryopreservation) should be considered with caution, since they can promote DNA clump formation and yield misleading concentration after thawing [173]. Safarikova et al. [173] tested DNA storage in liquid nitrogen and found it particularly unreliable, as preservation largely depends on operator handling during mixing and thawing. However, they noted that this result might be specifically related to their laboratory procedures, instead of universally shared [173].

- Desiccation

Desiccation is based on a mechanism used in nature by Tardigrades, which shift between hydrated and dehydrated states through trehalose. This discovery led to the adoption of desiccation as a protective strategy for DNA molecules in vitro [174]. Trehalose is one of the reagents used for dry storage (examples discussed in the next paragraph “Chemical preservation”), but desiccation can also be performed without reagents, and at different temperatures.

### 5.2. Chemical Preservation

- Trehalose

Trehalose is a disaccharide compatible with several preservation techniques, including dry storage (see previous paragraph “Physical preservation”), even though it is susceptible to degradation under temperature fluctuations or during prolonged storage. Smith and Morin [175] identified 0.2 M trehalose as an optimal medium for long-term DNA drying and storage at RT, yielding results comparable to those of the traditional storage in TE at −80 °C. Aly et al. [176] later confirmed this, reporting 100% PCR efficacy for trehalose-preserved DNA at both RT (22–38 °C) and −80 °C for up to 6 months. Trehalose can also be used as a protective matrix in other stabilization methods, including DNA shells [177]. However, in a more recent article [178], long-term storage at RT (temperature not specified) with trehalose was not found to be particularly effective. Nevertheless, the storage outcome was improved after raising the temperature at 56 °C, emulating accelerated aging conditions [178]. DNA preservation in trehalose also appears to be concentration-dependent, with higher concentrations provide greater protection against degradation [179].

- TE buffer (Tris-EDTA buffer)

Compared with water, TE buffer can slow DNA degradation by 1700 times [180]. Smith and Morin [175] used a TE buffer to preserve extracted DNA from human placenta and gorilla feces, measuring the quantity by qPCR after 12 months. The results were comparable to other preservation techniques (Lambda HindIII DNA (New England Biolabs, Ipswich, MA, USA, #N3012S) and trehalose) only in −20 °C and −80 °C (gorilla and placental DNA = 9.5–10.5 pg). The main difference was observed under dry storage, favoring trehalose over TE (gorilla DNA = 6.65 versus 11.55 pg; placental DNA = 3.5 versus 9.45 pg) [175]. Rathbun et al. [181] found that mtDNA extracted from saliva stored in TE at −20 °C, 4 °C and 20 °C was suitable to provide understandable data from massive parallel sequencing after 8, 22 and 34 weeks, respectively. The authors recommended lower temperature in combination with low-buffered alkaline (pH 8) TE for optimal stabilization [181].

- GenTegra^®^ (LLC, Pleasanton, CA, USA)

GenTegra^®^ is a matrix that uses the principle of desiccation. It stores DNA at RT for up to 6 months through drying. Frippiat et al. [182] applied it to forensic DNA samples in comparison with QIAsafe (QIAGEN, Germantown, MD, USA) (production discontinued). DNA recovery, in relation to the reference samples, is approximately 111% for GenTegra^®^, and 78% for QIAsafe. Overall, none of the two methods clearly outperforms the other [182]. The GenTegra^®^ matrix works well even with low DNA quantities, such as 3 ng [182] and between 1 ng and 0.2 ng [183]. Preservation periods longer than 6 months are also possible without damaging nucleic acid [183].

- SampleMatrix^TM^ (SM; Biomatrica, Inc., San Diego, CA, USA)

This matrix has been successfully used to preserve forensic DNA extracts at RT, even for long-term storage, and performs well in comparison to freezing [184].

- DNAstable^TM^ (Biometrica^®^, San Diego, CA, USA).

This reagent is a valid alternative providing successful DNA preservation at freezing temperature as well as RT [185]. The effectiveness is maintained for low-quantity DNA (<20 ng/µL) and without interfering with STR profiles. At high temperatures, DNAstable^TM^ slowed the process of degradation, as evidenced by lower Ct values compared to unprotected samples under the same conditions; however RT storage overall performed better [185]. Production has been discontinued.

- DNA/RNAse free water

Pajnič [186] demonstrated that DNA extracted from different types of human forensics and ancient samples (bloodstains, swabs, nails, bones) showed a statistically significant loss when resuspended in water. Even though the authors did not compare the data with DNA buffer-based storage, they recommended avoiding the use of water for long-term storage [186].

### 5.3. Emerging Storage Technology

Some innovative techniques have not yet been consistently applied in forensic and ancient genetics. Nonetheless, their potential should be acknowledged when considering future incorporation. These methods all relate to encapsulation techniques.

One of the most effective methods for storing dried DNA is DNAshell^®^ (Imagene, Pessac, France), which consist of hermetically sealed stainless-steel capsules. Through the process of DNA crystallization and subsequent splitting at temperatures between 100 and 140 °C, the probability of a break in DNA stored in the capsules was calculated to occur once every 100 years for every 100,000 bases. In other words, a 150 base pair (bp) fragment would be completely split in approximately 38,000 years at RT, three orders of magnitude superior to any alternative method [187].

Clermont et al. [177] demonstrated the efficacy of DNAshell^®^ by analyzing human DNA of varying quality after 18 months of storage and finding no decrease in DNA concentration. Accelerated aging results corresponding to 100 years of storage at 25 °C were equivalent for DNAshell^®^ and GenTegra^®^ in dried samples. However, DNAshell^®^ demonstrated significantly greater efficacy at 50% relative humidity (RH). Following one month of exposure to extreme temperatures, DNA concentration in the capsules reached 30–90% compared to the control, with trehalose required to achieve higher yields [177]. Coudy et al. [187] similarly tested DNAshell^®^ on DNA extracted from blood and accelerated aging, employing the Arrhenius equation. This study demonstrates the high level of DNA stability compared with other commercialized products by calculating the time required for a DNA molecule to degrade to 25 nucleotides, resulting in 1,070,000 years. Nonetheless, the accelerated aging models reported [177,187] represent extrapolations rather than empirically observed performance of DNA stability during storage.

Chen et al. [188] explored the potential of using capsules with magnetic nanoparticles interlaced with silica (SiO_2_) layers for stabilization. This approach yielded up to 3.4% of total capsule weight. In their experiment, most of the DNA was absorbed by nanoparticles after four assembled layers. Silica was applied and subjected to accelerated aging conditions (70 °C and 50% RH), after which it retained at least 89.5% of the 186 bp DNA intact. Silica encapsulation was also used by Narvaez Villarrubia et al. [189], who developed a one-step RT (temperature not specified) DNA recovery method only using a microwave, particularly suitable for long-term stabilization.

A novel proposal for encapsulation, inspired by biological samples preserved for millennia in minerals or amber, is called Thermoset-Reinforced Xeropreservation (T-REX) [190], based on deconstructable glassy thermosets for long-term storage of nucleic acids at RT. The authors consider it superior to silica and calcium phosphate encapsulation, as the presence of polyplexes allows nucleic acids of different lengths to pass directly from aqueous solution to organic solvents during polymer network formation, without the need for a drying step [190]. It is also worth mentioning the work of Elliott and Stotesbury [191], who tested the encapsulation of degraded DNA in alginate-based hydrogels. They showed that, even though DNA was successfully encapsulated, the degradation affected hydrogel rheology; shorter degraded DNA fragments produce weaker, less elastic gel [191].

## 6. Cross-Cutting Operational Factors

Among the chosen references, some differences stand out. First of all, most are peer-reviewed research articles, but other sources (Figure 1) also include reviews, commentaries, communications, reports, technical notes, and book chapters.

References are also classified in several categories according to the topic. The main part of the review is composed of references about human samples (47.0%), against 29.7% about other animal species (Figure 2). This result was expected, since the initial article search returned substantially more results for humans than for other species.

Another consideration to make is about the retrieval of references on forensic genetics and ancient DNA (Figure 3). Together they make up most of the article base, while the remaining 26.7% of references covers (i) general studies on DNA degradation mechanisms; (ii) general consideration about forensic genetics and ancient DNA; (iii) studies on the preservation of degraded samples or general sample preservation, when field-specific references were unavailable; and (iv) studies addressing the preservation of extracted DNA outside forensic or ancient DNA contexts. Including references that are examples of storage techniques not directly pertinent to forensic genetics or ancient DNA is still relevant. In fact, these references were retained to ensure representation of relevant sample types and to highlight gaps in the literature, particularly the limited representation of certain samples, as consistently observed throughout the review.

Below we present a preservation workflow spanning sample collection, transportation, and laboratory storage (Figure 4). At each stage, the appropriate strategy depends on a combination of logistical constraints (e.g., availability of a cold chain) and sample-intrinsic factors (e.g., tissue type, degradation state). As highlighted in Figure 4, freezing and drying/desiccation emerge as the two most broadly applicable preservation strategies across sample types, although their relative effectiveness varies: freezing is well supported under the tested conditions for long-term storage of mineralized tissues and blood, whereas drying-based approaches are generally adopted for keratinized structures, secretions, and field conditions lacking cold-chain access. The relative suitability of these strategies also depends on the downstream endpoint. Forensic workflows prioritizing interpretable STR/MPS profiles may tolerate different degradation thresholds than ancient DNA research aiming to maximize endogenous content and authenticate short, damaged fragments. Notably, for extracted DNA, cryopreservation and water storage are associated with risks of poor outcomes and should be carefully evaluated.

## 7. Discussion

DNA degradation in vertebrate samples must be considered at the early stages of research or sampling for ancient or forensic study purposes. As highlighted throughout this review, no single method suits every scenario. The appropriate preservation strategy depends heavily on the circumstances.

The outcome of genetic analysis is affected not only by the preservation conditions in the laboratory, but also by the sample conditions before sampling/excavation, and subsequent handling and transport. During excavation, specimens should be handled with gloves, and cleaned with a brush rather than washed [8]. It is also recommended to keep the samples cool, away from sunlight and heat exposure [8]. The easiest way to do this, in the absence of electric power, is to bury the samples in the ground [8]. Differences in excavation sites have been identified as factors influencing ancient genetic analysis [105]. Caves, for instance, offer optimal conditions for DNA recovery [8,42,192], primarily due to their stable low temperatures, since cold environments slow degradation [33], unlike higher-temperature and higher-humidity environments, which compromise DNA preservation.

Degradation is known to increase during post-excavation storage, such as in museums, where ambient conditions are highly variable [193], and temperatures and oxygen levels can be elevated [194] compared to archeological sites. Nonetheless, this degradation might diminish after a couple of decades, eventually becoming indistinguishable, as suggested by Gravlund et al. [193], who found no difference in the DNA damage rate of animal bones stored for 8 and 153 years under the same conditions.

The sampling phase should be performed with care and in accordance with the requirements for downstream analysis. We wish to highlight the star-based classification system proposed by Wong et al. [195] which, even though not designed for ancient and forensic samples, could be adapted with additional requirements for this type of degraded material. For the ideal characteristics of DNA preservation technology, Huang et al. [196] suggested high DNA loading capacity, stability, fast and gentle DNA release, low costs, accessibility, and the absence of toxicity.

As already discussed, collection timing is important, as DNA integrity progressively deteriorates with increases in the post-mortem period [21]. In addition, environmental conditions and intrinsic characteristics of the samples further influence nucleic acid preservation. Recently, Valsecchi et al. [197] offered an interesting perspective on the influence of the delay between defecation and collection of monk seal dung, which proved to be more relevant than the storage method itself. In case of early collection, fecal samples provided accurate genetic information even when stored in ethanol [197]. Although freezing during transport remains an option, possible temperature fluctuations may induce DNA degradation [198].

Taken together, our data suggest that dry storage, using FTA cards^®^, silica desiccants or other RT solutions such as DNA/RNA Shield™, can facilitate sample preservation during field sampling and transport while reducing the need for expensive cooling equipment and minimizing space requirements.

Standardized handling further protects physical integrity, minimizes cross-contamination risks, and supports legal admissibility in court. ISO 21043-2 (Forensic sciences—Part 2: Recognition, recording, collecting, transport and storage of items) [199] specifies requirements for the recognition, recording, collection, transport and storage of forensic items, including quality requirements, applicable both at the scene and within the facility. NIST/OSAC guidance (Organization of Scientific Area Committees for Forensic Science) complements this concept with more specific, discipline-based methodologies at the laboratory level.

Within the laboratory, establishing a clean environment and following careful protocols remains essential, especially to avoid contamination that could lead to repeated analyses and sample loss. For old specimens in particular, the contamination risk depends more on who handles washing and cleaning than simply on who collects the sample in the field [200].

Combining different storage techniques is often advantageous, since single methods might not satisfy all requirements. Salis et al. [198] demonstrated the efficiency of preserving field samples first using a buffer and then relying on cryopreservation. This technique should protect DNA integrity better than direct flash-freezing in liquid nitrogen, because buffer storage shields DNA from the freeze–thaw cycles that flash-frozen tissues undergo [198]. Nonetheless, according to the selected references, the most effective storage methods for the laboratory/archival settings are freezing and dry storage (especially at RT), both possibly coupled with a chemical storage solution. Preferred techniques also vary according to sample type. Liquid storage solutions like DMSO, modified TENT buffer, and LST buffer are frequently used for soft tissues, although their relative performance depends on storage duration. DMSO and NaCl are effective across both short- and long-term storage, whereas TENT buffer and FTA^®^ cards perform adequately only on fresh samples and lose effectiveness within approximately two months. FTA^®^ cards are the most common choice for blood samples. Bones show a duration-dependent preference, being better preserved in ethanol for short periods, while freezing is common for longer periods. Freezing or RT storage are mostly employed for teeth and tusks, without a clear dependence on the storage method or duration. Saliva can be stored dry or in liquid preservatives at RT. Among keratinized structures, hair is well studied and is generally stored dry at RT, with efficacy also over the long term. Other keratinized structures, such as nails, claws, hooves, scales and feathers, remain underrepresented in the forensic and ancient DNA literature, even though dry storage and, where available, freezing are mostly common. Data are even more limited for urine, dung, touch DNA, and swabs; although they are frequently stored dry, the available information is insufficient to provide more specific indications. Consequently, this review cannot establish an optimal storage strategy for each biological source of DNA. However, even for those samples that are underrepresented in terms of storage studies, each of them remains a valuable source of DNA, and suitable preservation methods are worth investigating. Hair, in particular, is a precious, persistent source of DNA that should be sampled and tested whenever available. Further details supporting these observations and offering a more comprehensive overview are provided in the Supplementary Material (Table S1).

Other factors influencing DNA degradation include sample purity, tube type, ionic strength [201], and choice of an appropriate extraction method, which should be tailored to the sample to maximize DNA yield and minimize sample loss. For extracted DNA storage, freezing remains widely used, but attention must be paid to limit the number of freeze/thaw cycles; therefore, making aliquots is fundamental. Desiccation, especially with trehalose, is effective for both short- and long-term storage, although findings across studies are not fully consistent. Moreover, interesting emerging techniques of encapsulation, like DNAshell^®^, magnetic nanoparticles and T-REX, might be applicable in the future for forensic and ancient samples.

Overall, based on the available data, the storage methods presented do not show clear differences between humans and other vertebrate samples, although species-specific biological factors, such as nucleated avian blood [30] and the absence of bone remodeling in fish [105], can influence preservation dynamics for specific sample types. It should also be noted that some sample types, such as feathers, scales, tusks, and hooves, obviously have no direct human equivalent, making direct comparison limited. Moreover, much of the animal sample literature reflects field-driven, non-invasive collection under logistically constrained conditions, rather than the controlled laboratory settings more typical of human forensic samples. Finally, the depth of the comparative literature is unevenly distributed across taxa and sample types.

It is in our interest to acknowledge Singh [202], whose study shares the objective of summarizing the best practices in forensic sample storage, covering the workflow from collection to laboratory storage. Although our review adopts a different approach, Singh’s work provides a valuable basis for comparison with the framework proposed here.

As already discussed, preservation strategies require careful evaluation in terms of analytical endpoints. Notably, high DNA recovery is not necessarily related to successful downstream analysis and long-term preservation, as shown in several examples [69,156,165]. Conversely, Lacerenza et al. [164] demonstrated that lower DNA yield is not always translated into worse STR profiling outcomes; in their experimental setup, the lower recovery obtained with ethanol did not affect STR completeness, since the amount of DNA recovered allowed them to retrieve complete STR profiles. Similarly, Higgins et al. [110] illustrated this principle within teeth; nDNA yield was highest in cementum, while mtDNA yield was highest in root dentine, yet STR typing success followed the nDNA pattern. This shows that DNA yield can be misleading unless the specific target and downstream application are defined. The recovered DNA quantity can also fail to reflect other properties related to downstream use, as shown by Thornton et al. [106], who found comparable DNA quantities across different samples and environments, but significant differences in DNA quality. These findings illustrate the complex relationship between recovery and downstream applications. Higher DNA recovery remains desirable and informative, but because samples and sampling strategies can differ and may affect DNA recovery, relying too heavily on yield to assess the suitability of a preservation strategy is not appropriate. Recovery, preservation, and downstream analytical performance should instead be evaluated and reported as distinct outcomes, each providing complementary but non-interchangeable information.

Forensic and ancient DNA workflows are often discussed under a single DNA degradation framework, yet the endpoints they optimize for differ substantially. Forensic casework prioritizes the recovery of interpretable STR/MPS profiles, prevention of contamination and PCR inhibition, and sample traceability for potential re-analysis, reflected in the widespread reliance on validated collection media and buffers [22,74,76] and in the documented risks of cross-contamination in bone-based casework [199]. Ancient DNA research instead prioritizes maximizing the endogenous DNA fraction, recovering ultra-short and chemically damaged fragments, characterizing degradation patterns, and establishing sequence authenticity against modern contamination [17,18,19,108]. Higgins et al. [110] illustrated why these priorities cannot be treated as a single preservation endpoint: nDNA and mtDNA showed markedly different preservation trajectories within the same post-mortem teeth, with direct but divergent implications for forensic STR typing versus ancient DNA endogenous recovery.

## 8. Conclusions

Multifactoriality is an essential aspect of DNA degradation. This is particularly challenging when facing samples with low quality and a limited quantity, such as ancient and forensic samples. No preservation method is perfect, since several factors influence outcomes, including the sample type and downstream applications, which should be taken into consideration, including whether the analytical goal is forensic or ancient DNA, as these pursue markedly different endpoints. However, according to the selected references, some preservative methods are more common than others. As already stated, freezing, ethanol, formalin, and formaldehyde have traditionally been used as preservatives for all kinds of specimens, even though associated downsides are known and cannot be ignored. According to the collected data, freezing and RT-based dry storage remain the most common approaches overall, with liquid storage solutions next, but their performance is strongly sample-dependent. RT storage is advantageous in terms of cost and space during transport, when cold-chain access is limited. Freezing remains common for long-term storage. Nonetheless, studies show that combining more strategies together can lead to more success in genetic analysis by improving DNA yield, PCR amplification and STR typing success. For the storage of samples like urine, dung, touch DNA, and swabs, the available evidence remains insufficient to draw definitive conclusions; however, dry storage appears to be a commonly used approach.

## Figures and Tables

**Figure 1:**
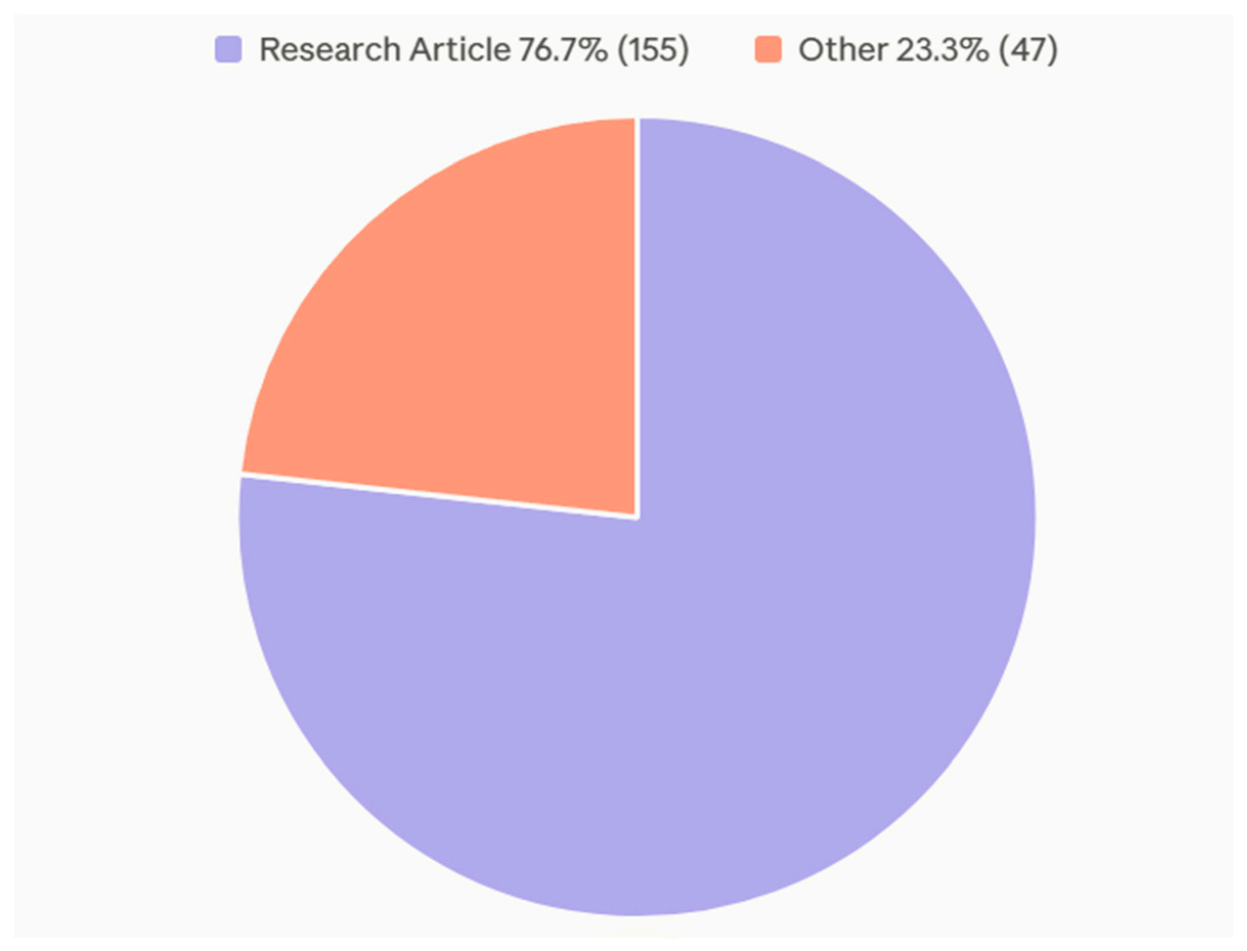
Distribution of publication types among the 202 references included in this review. Articles classified as “Other” (n = 47) comprise reviews, book chapters, case reports, technical notes, short communications, and similar non-original-research formats.

**Figure 2:**
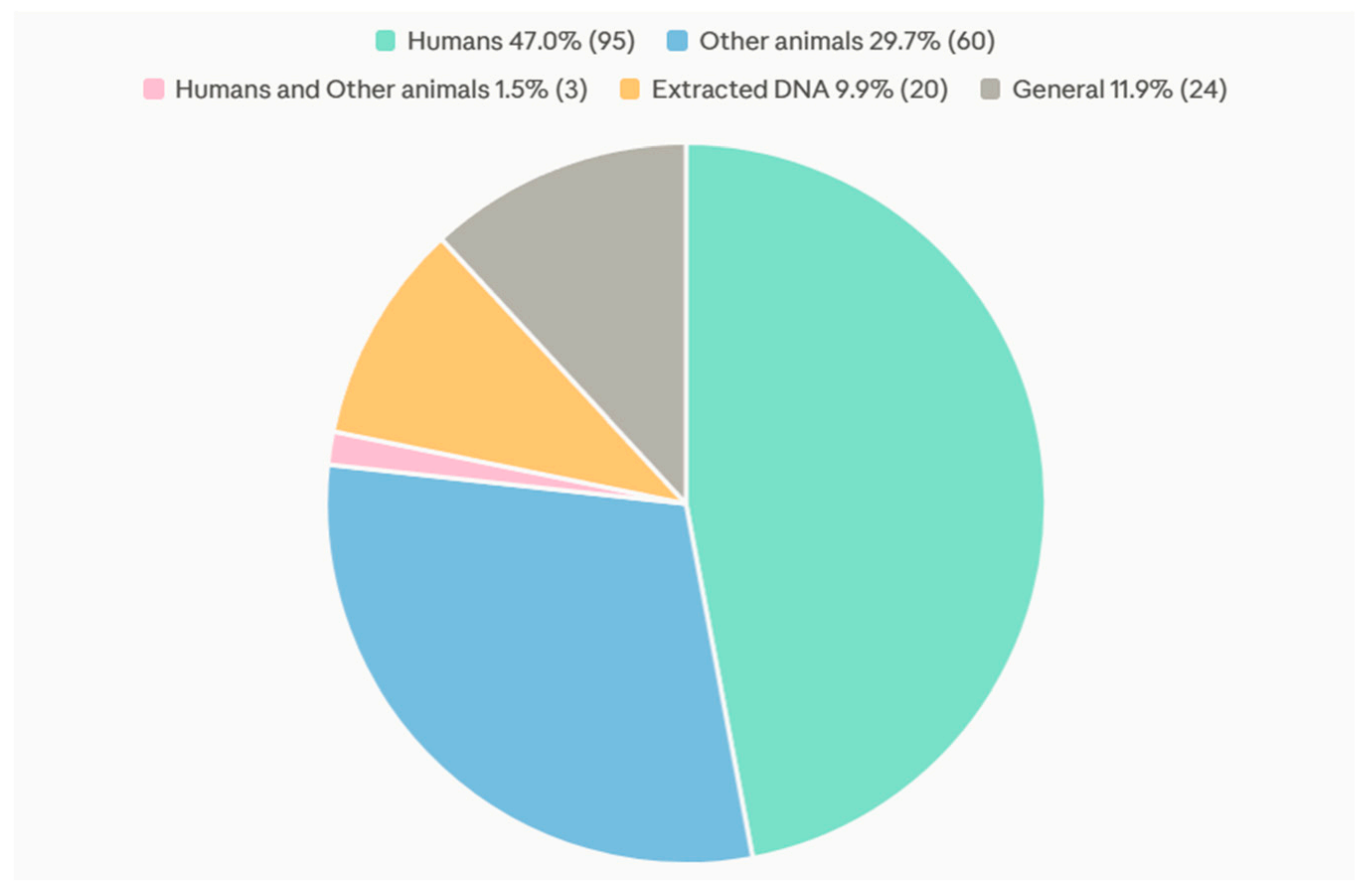
Distribution of the 202 references according to the theme. References were classified as “Humans” (n = 95) or “Other animals” (n = 60) when the study focused on samples from one of these groups; “Humans and Other animals” (n = 3) when both were addressed; “Extracted DNA” (n = 20) when the study concerned DNA already extracted rather than a specific biological sample; and “General” (n = 24) when the reference addressed DNA degradation mechanism and storage techniques broadly, without reference to a specific sample type or species.

**Figure 3:**
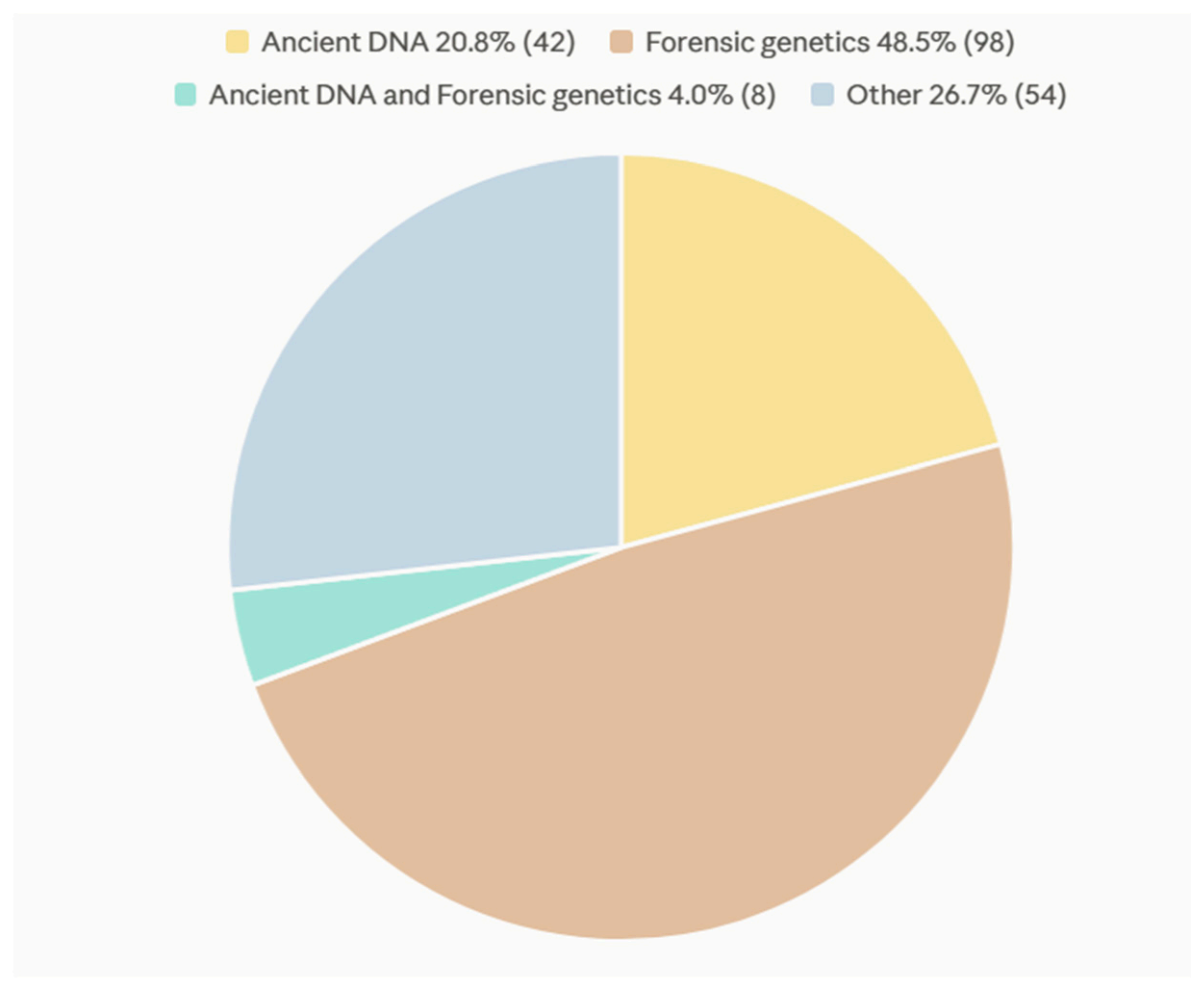
Distribution of studies according to the specific topic of the review, where “Ancient DNA” refers to specific studies on ancient samples; “Forensic genetics” means studies on forensic samples; “Ancient DNA and Forensic genetics” comprises studies explicitly addressing both fields; and “Other” includes studies not specifically focused on ancient or forensic context or other general documents regarding the presented topic.

**Figure 4:**
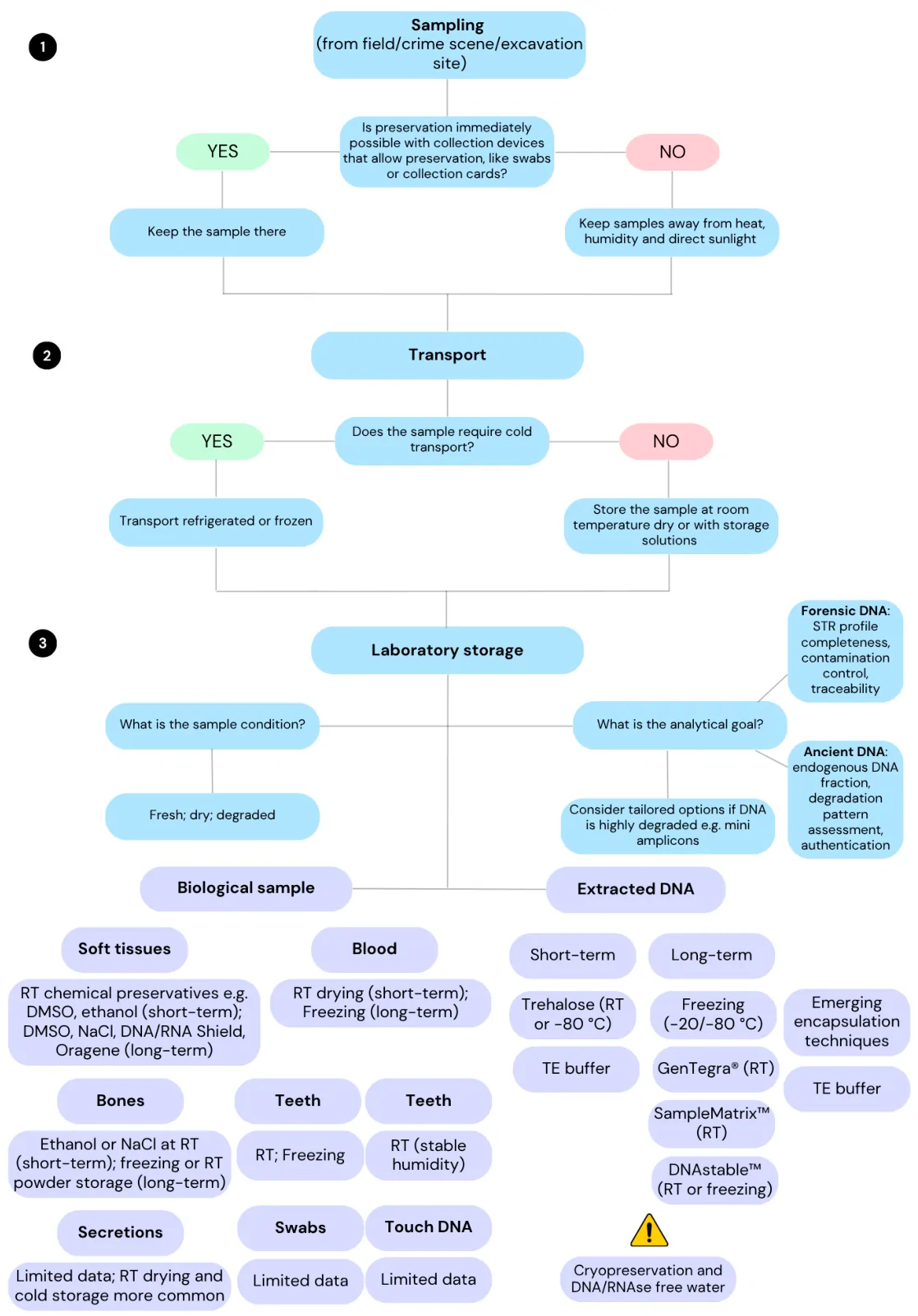
Workflow for the preservation of biological samples from sampling (1) to transport (2) and laboratory storage (3).

## Data Availability

No new data were created or analyzed in this study. Data sharing is not applicable to this article.

## Supplementary Materials

The following supporting information can be downloaded at: https://www.mdpi.com/article/10.3390/life16101601/s1, Table S1: The information summarized in this table reflects findings as reported in the specific studies reviewed and should not be interpreted as exhaustive or universally applicable recommendations. Sample types, temperatures, and durations listed for each storage method correspond to the conditions tested in the cited literature; a given method may be effective, or already in use, for additional sample types or storage conditions not covered by the available evidence. This table is intended as a synthesis of documented performance rather than a definitive protocol.

## Abbreviations

The following abbreviations are used in this manuscript:

mtDNA	Mitochondrial DNA
nDNA	Nuclear DNA
aDNA	Ancient DNA
NGS	Next Generation Sequencing
RT	Room Temperature
