# Supplementary material for: DNA Preservation in Vertebrate Samples: Insights from Forensic and Ancient Contexts
Source: Life (Basel). 2026 Sep 24;16(10):1601. doi: 10.3390/life16101601 (PMC13705696; doi:10.3390/life16101601)
Supplement: Supplementary file 1 [file life-16-01601-s001.zip › life-4526370-supplementary.pdf]

Storage method	Sample	Temperature	Duration	Analytical endpoint	Key result	Principal advantage	Principal limitation	Field suitability	References
**Refrigeration**	Blood, urine, extracted DNA	+4 °C	Short-term/long-term (variable by sample)	DNA yield; degradation index; STR profile	Effective for urine, with STR typing results similar to –20°C storage and long-term preservation already demonstrated decades ago; considerably less reliable for blood-based matrices; a blood-soil mixture stored at 4°C showed a rapidly rising degradation index and no detectable DNA by week 8, and pure bloodstains showed lower integrity than -20°C storage over 20 years	Simpler cold-chain requirement than freezing	Inconsistent across sample types; unsuitable for some matrices such as blood-soil mixtures	Moderate. A portable refrigerator is required	[47,69,70,134,143,144]
**Freezing**	Soft tissues, blood, bone, urine,	-20 °C<br>-80 °C	Long-term	Fragment integrity ratio; DNA yield; STR profile	Highest integrity for blood achieved at ≤–20°C over	Broadly applicable across sample types; slows	Requires a constant cold chain; freeze/thaw	Moderate. A portable freezer is required	[45,70,77,83,84,87,88,106,143,165,172,173]

	extracted DNA				20 years; best 1-year performance for bone vs. RT/ethanol, but 10-year storage caused a ~5-fold yield decrease; extracted DNA is sensitive to repeated freeze/thaw cycles, with larger fragments degrading faster	enzymatic and chemical degradation effectively	cycling degrades DNA; space and energy are required		
**Cryopreservation**	Extracted DNA	≤–80°C	Long-term	DNA clump formation; post-thaw concentration accuracy	Found less reliable than standard –80°C storage; leads to DNA clump formation and potentially misleading concentration measurements after thawing; outcome may depend on operator handling	Near-complete halting of enzymatic activity in principle	Requires a constant cold chain; risk of DNA clumps; space and energy required	Low. Specialized equipment is required	[173]

**Dry storage**	Soft tissues, hair, teeth, feces	RT Freeze-drying temperature	Short/long-term	STR profile; DNA yield	Silica desiccation performed comparably to freezing for hair; freeze-drying yielded ~2x more DNA than air-drying in teeth; silica preservation outperformed other tested methods for bear feces	No cold chain required; low cost; simple field application	Effectiveness varies by drying method (freeze-dry vs. air-dry) and by species	High. Well suited to remote/field collection without refrigeration	[48,116,118,133,151]
**Emerging technologies (Encapsulation)**	Extracted DNA	RT +4 °C -20 °C	Long-term	Accelerated-aging extrapolation; DNA concentration retained	DNA shells projected to have extreme stability (a 150 bp fragment modeled to fully split in ~38,000 years at RT; a separate Arrhenius-based estimate projected ~1.070.000 years	High DNA stability; high DNA retrieval	The applications are still largely focus on accelerated-ageing studies rather than empirical studies; expensive equipment; consuming sample preparation	Not required because it is used for extracted DNA	[177,187,188,189,190,191]

to degrade to 25 nucleotides); magnetic nanoparticle/silica capsules retained 89.5% of 186 bp DNA after accelerated aging; alginate hydrogels successfully encapsulated DNA, though degraded fragments reduced gel elasticity; 18-month storage showed no DNA loss (empirical result); other encapsulation methods (microwave-silica, T-REX) are also promising for

					long-term RT storage				
**Non-commercial reagents:**									
Isopropanol	Soft tissues	Not specified	Short-term	Amplification success	Provided good short-term preservation for pig soft tissue in a comparative study, performing comparably to ethanol and outperforming desiccation in the same test.	Wdely available; cheap	Data limited to a single comparative study; long-term performance not assessed	Moderate. Usable for short-term field storage	[50]
NaCl	Soft tissues, bones	RT	Long-term	DNA recovery; STR profile	Preserved tendons with ~100% DNA recovery vs. 3% untreated; soft tissue in dry salt for >1 year showed no damage and outperformed –20°C storage; bone preserved	Low cost; RT-stable; widely available; cheap	Modern experimental data are mostly limited to periods under 1–2 years	High. No cold chain needed, simple and field-compatible	[56,61,62,63,89,90]

					30 days at RT without affecting DNA quality; historically used in ancient mummification.				
DMSO	Soft tissues	RT<br>+4 °C<br>-20 °C	Long-term	Amplification success rate at defined amplicon length	Most effective of several preservatives tested on pig tissue, with >90% amplification success for a 642 bp amplicon over 6 months even in degraded samples; remained effective at high ambient temperature (tropical DVI conditions)	Performs well at high ambient temperature; suited to tropical/remote DVI contexts	Requires correct reagent handling; less studied outside DVI-type contexts	High. RT-stable, suitable for field/DVI collection without cold chain	[48,50,55,56]
DESS	Soft tissues	RT<br>+4 °C<br>-20 °C	Long-term	Degradation rate over time	Mild degradation after 3 months	Stabilizes DNA via deactivation	Limited long-term reliability not established	High. No cold chain needed, simple and field-compatible	[43,47]

					vs. high degradation after 24 hours with ethanol in avian and fish tissue; not always reliable long-term, retaining only ~50% high-quality DNA even when combined with freezing	of metalloproteins/DNases; performance comparable to cryopreservation; can be further improved when combined with refrigeration	beyond a few months		
TE (Tris-EDTA) Buffer	Extracted DNA	RT<br>+4 °C<br>-20 °C	Long-term	DNA yield (pg); mtDNA sequencing usability	Slows degradation up to 1700x vs. water; yield comparable to trehalose only at –20/–80°C, while trehalose outperforms it at RT/dry state; low-buffered alkaline TE (pH 8) with cold storage gave usable MPS data after 8–34 weeks	Widely available; cheap	The presence of EDTA can interfere with downstream analysis	Not required because it is used for extracted DNA	[175,180,181]

Modified TENT	Soft tissues	RT	Short-term	STR profile	Yielded higher DNA quantity and more complete STR profiles than FTA® cards on decomposing tissue in one study; however, produced mostly partial profiles despite high DNA yield in fresh tropical-condition tissue and in decomposing tissue over 3 months at 35°C (58% partial); more suitable for fresh tissue overall, with low preservation potential after 2 months	Widely available; cheap	Effectiveness declines rapidly with tissue degradation and storage time	High. No cold chain needed, simple and field-compatible	[57,58,59]
NAP buffer (Nucleic Acid Preservation Buffer)	Soft tissues	RT	Short-term	Onset of degradation; comparative preservation	Outperformed 95% ethanol and cryopreservation over 7 weeks in postmortem	Cheap, homemade, outperformed common alternatives	Limited long-term data; degradation still	High. No cold chain needed, simple and field-compatible	[67]

					tissue; EDTA content credited for improved preservation; degradation was still detectable from 6 hours postmortem	in short-term testing	begins quickly postmortem		
Lysis Buffer	Blood, saliva, touch DNA	+50 °C RT	Short-term	DNA integrity after set exposure; field DNA yield	More efficacious than distilled water for touch DNA (undamaged after 24h at 50°C vs. degraded in water); successful predator-saliva retrieval from field kill sites even with very low remains (logistical choice of the buffer)	RT-stable, effective for low-template and field samples	Limited comparative data across sample types	High. No cold chain needed, simple and field-compatible	[83,157,167]
Longmire's Lysis Buffer	Soft tissues	+60 °C	Short-term	DNA integrity; reproducibility of amplification	High DNA integrity maintained for 3–4 weeks at 60°C, but no	Maintains DNA integrity under high-temperature	Downstream effectiveness cannot be assessed from available data	High. No cold chain needed, simple and field-compatible	[66]

					reproducible amplification results were obtained, so downstream effectiveness could not be confirmed	stress conditions			
Lysis, Storage and Transportation Buffer (LST)	Soft tissues	RT	Long-term	DNA yield; STR profile completeness	Outperformed by the Oragene kit in overall DNA yield, but STR profile quality was not significantly different; effective for preserving low tissue amounts (<100 mg); degradation began after 6 months	More effective than Oragene for small/limited tissue samples; RT storage	Lower overall DNA yield compared to the Oragene® kit for standard/larger tissue amounts; degradation still begins after ~6 months, similarly to Oragene	High. No cold chain needed, simple and field-compatible	[60]
Forensic DNA Laboratory Buffer (FDL Buffer)	Blood, saliva	RT	Short-term/long-term (variable by sample)	DNA yield	4-month-old blood samples stored at RT with FDL buffer yielded ~2.6 µg of DNA,	RT-stable; outperformed sodium citrate for blood in the	Yield is strongly sample-type dependent (markedly lower for saliva); data limited to a	Moderate. RT storage feasible, but performance is highly sample dependent	[71,154]

					compared to ~2 µg with sodium citrate; FDL buffer tested on saliva yielded a much lower amount than on blood; a self-made FDL buffer separately proved effective for preserving saliva for years at RT	tested comparison	single medium-term time point		
Trehalose	Soft tissues, extracted DNA	+56 °C<br>RT<br>-80 °C	Long-term	Amplification success; DNA concentration retained	0.2 M trehalose performed comparably to TE buffer at –80°C for long-term RT storage; 100% PCR efficacy demonstrated at RT and –80°C over 6 months; higher concentrations improve protection, but some studies found long-term	Enables RT storage without a dedicated cold chain; combinable with other matrices; cheap	Efficacy inconsistent across studies; concentration-dependent; the presence of EDTA can interfere with downstream analysis	High. Supports RT/field-compatible extracted-DNA storage	[68,175,176,177,178,179]

					RT storage ineffective unless combined with elevated temperature (56°C)				
Ethanol	Soft tissues, hard tissues, dung	RT<br>+4 °C<br>-20 °C<br>-80 °C	Short-term	Amplification success; DNA degradation rate	Provided good short-term preservation for pig tissue; 90% ethanol was the best of several methods tested for bear dung; showed a much higher degradation rate than DESS in avian and fish tissue within just 24 hours; 70% ethanol preserved DNA in muscle tissue for 2 weeks at 37°C and up to 4 weeks at RT; outperformed by a home-made NAP buffer over 7 weeks in	Widely available; cheap; simple to use	Recommended concentration (70%) must be respected; possible inhibition of downstream analysis; poor long-term reliability in some tissue types (e.g., avian, fish) compared to dedicated buffers	Moderate. Field-usable but concentration must be controlled	[43,47,50,56,67,83,150]

					postmortem tissue				
EDTA	Blood, urine	+4 °C<br>-20 °C<br>-80 °C	Long-term	STR loci detection rate	In blood, EDTA was used solely as an anticoagulant without additional preservative, and degradation patterns did not correlate consistently with storage temperature; for urine, 40 mM EDTA at –80°C was identified as the most effective concentration for STR loci detection; EDTA content in other buffers (e.g., NAP buffer) is credited with improving preservation efficacy when	Simple, low-cost additive; improves preservation efficacy when combined with other reagents/cold storage	Concentration-dependent; not an effective standalone method without adequate temperature control	Moderate. Useful as an additive, but requires combination with proper storage conditions	[44,67,69,145]

					combined with them				
DNA/RNAse free water	Extracted DNA	RT<br>+4 °C<br>-20 °C	Short-term	Comparative DNA loss vs. buffer storage	Diluted DNA degraded faster; DNA from various human forensic and ancient sample types showed a statistically significant loss when resuspended in water compared to buffer-based storage	Not enough information	Not recommended for long-term because of low DNA stability	Not required because it is used for extracted DNA	[186]
**Commercial kits:**									
DNAgard®	Soft tissues	35 °C	Short-term	Genotype / STR profile completeness	Among eight reagents tested for tropical DVI conditions, DNAgard® was one of only a few (alongside DMSO and the DNA Genotek kit) to produce	Effective at high ambient temperature; versatile across solid and liquid preservation formats	Commercial reagent; comparative data currently limited to a single tropical DVI study	High. Validated specifically for tropical/remote DVI field conditions	[55]

					complete genotype/STR profiles in both solid and liquid preservation formats				
Allprotect™	Teeth	RT	Short-term	DNA quantity	Significantly increased DNA quantity in intact tooth samples stored at RT for up to 180 days (p=0.008), but provided no benefit for refrigerated or frozen samples, or for dental pulp under either temperature condition	Effective specifically for RT storage of teeth when refrigeration/freezing is not feasible	High cost. Justified mainly when extended RT storage is unavoidable; freezing remains the cheaper alternative when cold chain is available	Moderate. Useful for RT scenarios, but not needed if cold chain is accessible	[117]
GenTegra®	Extracted DNA	RT	Long-term	DNA recovery	Recovery efficiency ~111% relative to reference vs. 78% for QIAsafe; effective even at	RT storage via desiccation; effective at very low	Commercial cost; direct long-term head-to-head data still limited	Not required because it is used for extracted DNA	[182,183]

					very low DNA input (down to 0.2 ng); preservation confirmed beyond 6 months	DNA quantities			
SampleMatrix™	Extracted DNA	RT	Long-term	DNA recovery	Successfully preserved forensic DNA extracts at RT, including long-term storage, comparing favourably to freezing	RT-stable alternative to freezing for extracted DNA	Limited independent comparative studies available	Not required because it is used for extracted DNA	[184]
DNA/RNA Shield™	Soft tissues	RT	Long-term	STR profile completeness	Recognized as effective for long-term storage and better suited to already-degraded samples compared to modified TENT buffer	Effective specifically on degraded tissue; RT-stable	Commercial cost; comparative data still limited	High. RT-stable, field-compatible	[59]

DNAstable™	Extracted DNA	-20 °C, RT	Long-term	Ct value comparison; STR profile interference	Effective at both freezing and RT temperatures; maintained performance for low-quantity DNA (<20 ng/μL) without interfering with STR profiles; slowed degradation at high temperature (lower Ct vs. unprotected), though RT alone still performed best overall; production has been discontinued	Effective across a range of temperatures; compatible with low-quantity DNA	Product discontinued; RT storage alone was often as good or better	Not required because it is used for extracted DNA	[185]
FTA® cards	Soft tissue, blood, urine, saliva, touch DNA	RT	Long-term	STR profile completeness; degradation index (DI)	61.5% of 11-year saliva samples had an acceptable DI for STR typing; overloading introduces excess haemoglobin	RT storage, no cold chain, validated across many sample types, easy field/transport use	Sample overloading risk; species-specific issues (e.g., avian blood needs dilution); inconsistent	High. Among the most field-friendly, widely used methods across sample types	[56,57,74,75,76,78,81,141,142,156,168]

					causing PCR inhibition; unsuccessful for muscle tissue in one study (53% PCR-inhibited); double FTA scraping doubled touch-DNA yield vs. swabbing, though double swabbing retained a nominally higher (non-significant) STR allele recovery rate (99% vs. 91%); on concrete, FTA elution cards achieved ~92% STR success vs. ~17% for swabbing		performance on some soft tissues		
Oragene™ DNA self-collection kit	Soft tissues, saliva	RT	Long-term	DNA yield; STR profile completeness	Oragene outperformed LST buffer in yield from muscle tissue	RT-stable, simple self-collection format	Degradation still occurs after ~6 months; larger tissue input is	High. Designed for self-collection and remote/field use	[60,152,153]

					(optimal at 500 mg), with similar STR quality; LST performed better for small tissue amounts (<100 mg); degradation began after 6 months in both cases; saliva remained amplifiable after months to years at RT		required for higher yield		
RNA*later*®	Blood, swabs	RT<br>-20 °C<br>-80 °C	Short/long -term	Comparative DNA quality	Provided high-quality DNA for avian blood storage; has been reported to interfere with subsequent DNA extraction in some protocols; On mock skin stains, RNAlater® and	RT-stable stabilizing solution; suppresses RNase activity	Possible interference with DNA extraction	Moderate	[50,83,164]

ethanol produced significantly more complete STR profiles than water (>98% vs. 71%), though for other tissue types (blood, semen) yield differences did not translate into STR success differences

Table S1. The information summarized in this table reflects findings as reported in the specific studies reviewed and should not be interpreted as exhaustive or universally applicable recommendations. Sample types, temperatures, and durations listed for each storage method correspond to the conditions tested in the cited literature; a given method may be effective, or already in use, for additional sample types or storage conditions not covered by the available evidence. This table is intended as a synthesis of documented performance rather than a definitive protocol.
